# Simultaneous detection and estimation in olfactory sensing

**DOI:** 10.1101/2025.11.01.686013

**Published:** 2025-11-03

**Authors:** Chen Jiang, Matthew Y. He, Venkatesh N. Murthy, Cengiz Pehlevan, Jacob A. Zavatone-Veth, Paul Masset

**Affiliations:** 1 Department of Psychology, McGill University, Montréal, QC, H3A 1G1, Canada; 2 Quantitative Life Sciences, McGill University, Montréal, QC, H3A 1G1, Canada; 3 Center for Brain Science, Harvard University, Cambridge, MA, 02138, USA; 4 Department of Molecular and Cellular Biology, Harvard University, Cambridge, MA, 02138, USA; 5 Kempner Institute for the Study of Natural and Artificial Intelligence, Harvard University, Cambridge, MA, 02138, USA; 6 John A. Paulson School of Engineering and Applied Sciences, Cambridge, MA, 02138, USA; 7 Society of Fellows, Harvard University, Cambridge, MA, 02138, USA; 8 Mila - Québec AI Institute, Montréal, QC, H2S 3H1, Canada

## Abstract

The mammalian olfactory system shows an exceptional ability for rapid and accurate decoding of both the identity and concentration of odorants. Previous works have used the theory of compressed sensing to elucidate the algorithmic basis for this capability: decoding odor information from the responses of a restricted repertoire of receptors is possible because only a few relevant odorants are present in any given sensory scene. However, existing circuit models for olfactory decoding still cannot contend with the complexity of naturalistic olfactory scenes; they are limited to detection of a handful of odorants. Here, we propose a model for olfactory compressed sensing inspired by simultaneous localization and mapping algorithms in navigation: the set of odors that are present in a given scene, and the concentration of those present odors, are inferred separately. To enable rapid inference of odor presence in a biologically-plausible recurrent circuit, our model leverages the framework of Mirrored Langevin Dynamics, which gives a general recipe for sampling from constrained distributions using rate-based dynamics. This results in a recurrent circuit model that can accurately infer presence and concentration at scale and can be mapped onto the primary cell types of the olfactory bulb. This framework offers a path towards circuit models—for olfactory sensing and beyond—that both perform well in naturalistic environments and make experimentally-testable predictions for neural response dynamics.

## 1 Introduction

Animals sense the physical properties of their external world using specialized neural circuitry [1]. To enable rapid adaptation of behavior to the demands of a changing world, early stages of sensory processing leverage the statistical properties of sensed signals [2–5]. This evolutionary adaptation leaves an imprint on the structure of neural circuits [5–7].

**Figure 1:**
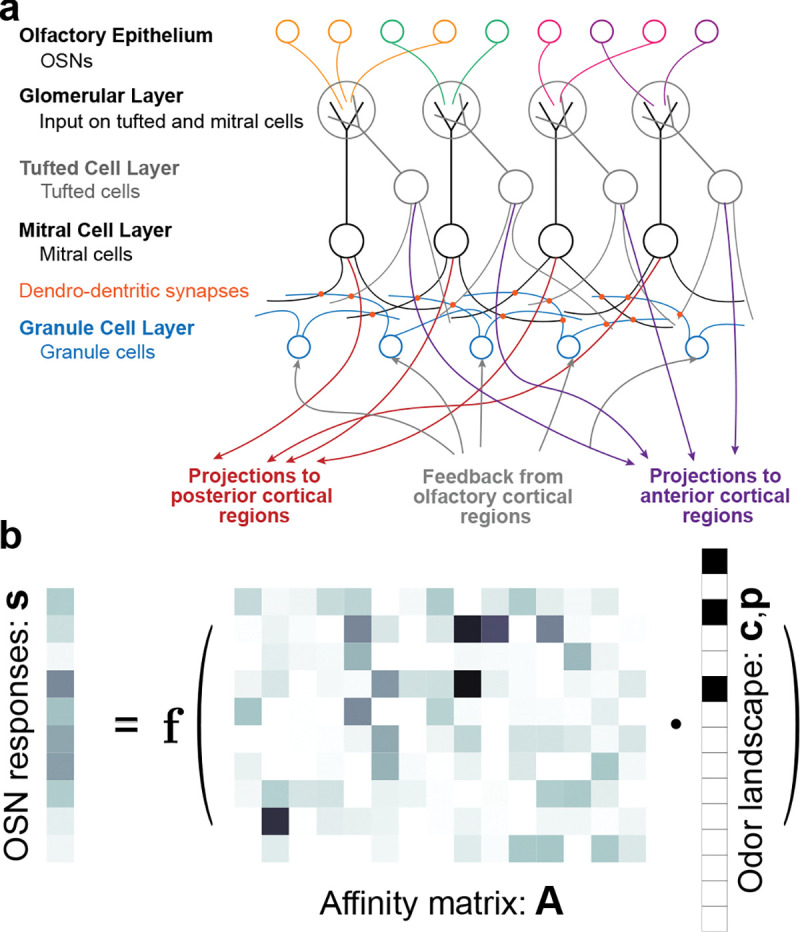
The structure of olfactory sensing. **a.** Outline of the anatomy of the mammalian olfactory bulb (OB). Volatile odorant molecules first bind to olfactory receptors on the surface of the olfactory sensory neurons (OSNs) that tile the olfactory epithelium. Each OSN expresses a single receptor type; humans have a repertoire of about 300 receptor types, while mice have about 1000 [9, 16, 43] OSNs synapse onto the primary excitatory projection neuron types of the OB—mitral and tufted cells—in clustered olfactory glomeruli containing axons of OSNs expressing the same, single receptor type [44, 45]. Mitral and tufted cells then project to higher areas, notably including the posterior piriform (primary olfactory) cortex and the anterior olfactory nucleus (AON), respectively [22, 25, 46, 47]. Recurrent connectivity within the mitral and tufted cell layers is mediated via inhibitory granule cells, which make dendro-dendritic synapses with their excitatory partners. Feedback from higher areas to the bulb comes in the form of synapses onto the granule cells. **b.** Dimensionality of the sensing problem. The compressed OSN representation **s** of the olfactory world (*left*) is given by a (stochastic) function f(·) of the product of the matrix **A** of the affinities of each OSN to each odorant (*center*) with the sparse, high-dimensional odor scene vector giving the concentrations of which out of millions of possible odorants are present in a given scene (*right*). For illustrative purposes, we show 10 receptors and 15 odorants.

The most important statistical analysis problem faced by many mammals is that of odorant detection, of analyzing the composition of a given mixture of scents. Mammals—particularly rodents—rely on reliable olfactory sensing to perform tasks essential for survival, such as detecting predators [8], recognizing conspecifics [9, 10], and locating food sources [11, 12]. However, olfactory sensing is a highly challenging computational task, as an animal has access only to intermittent samples of odorants wafted on turbulent plumes of air, from which it must determine which among a myriad of possible sources is present [11, 13–15]. This challenge is exacerbated by the fact that olfactory sensing is bottlenecked by the limited repertoire of olfactory receptor proteins, which range in number from ~ 300 in humans to ~ 1000 in mice (Figure 1a–b) [16]

Despite this challenge, rodents can perform many olfactory sensing tasks with exquisite speed and accuracy, often within the hundred-millisecond timescale of a single sniff [12–14, 17–19]. Due to the difficulties inherent in generating tunable yet naturalistic olfactory stimuli in the laboratory, detailed probes of these capabilities have only become possible in recent years [12, 14, 18–20]. This growing body of work has begun to elucidate the key role of the statistics of olfactory landscapes in perception and detection. Moreover, it has revealed that the different cell types of the olfactory bulb (OB)—the locus of early olfactory processing in mammals (Figure 1a)—display strikingly diverse tuning and dynamics in response to odor stimuli [21–25].

How might these remarkable abilities arise? A growing body of theoretical work suggests that the answer may lie in the theory of compressed sensing [26, 27]: accurate analysis is possible because only a few odorants—tens or hundreds out of a million or so possibilities—are present in a given scene [3, 28–40]. Given a compressed sensing framework, the uniquely-structured recurrent circuits of the OB offer a potential substrate for the implementation of simple algorithms to solve the resulting problem of inferring what odorants are present given the noisy activity of olfactory sensory neurons (OSNs) (Figure 1a). However, the algorithms proposed by these past works—including our own [39]—have not shown how to simultaneously overcome two key challenges: First, to generate experimentally-testable predictions, it is useful to show that the algorithm can be implemented as a biologically-plausible recurrent neural network (RNN). Second, the algorithm should be able to scale up to dynamic olfactory scenes with a naturalistically large odorant repertoire.

In this work, we propose a framework for olfactory sensing that allows us to achieve high detection capacity in a recurrent circuit model. Our model combines two central ideas: First, odorant presence and concentration are inferred separately. This split inference procedure is inspired by simultaneous localization and mapping (SLAM) algorithms in vision-guided navigation [41], and builds on previous works that have used split inference in olfaction to specify more flexible priors on presence [29, 36]. However, inference of odor presence is challenging to implement in a rate-based recurrent circuit because presence is binary; past works did not overcome this obstacle. This leads to the second main idea of our model: we leverage the methodology of Mirrored Langevin Dynamics [42] to design a recurrent circuit sampler that samples a softened presence estimate while remaining biologically plausible. This approach to designing simple recurrent circuits that can solve constrained inference problems should be applicable to sensory neuroscience beyond the context of olfaction.

We show that the resulting model enables fast and robust inference of more odorants than structurally-similar recurrent circuit models that do not split the inference problem, accurately analyzing static scenes with hundreds of odorants present out of a repertoire of tens of thousands within the timescale of a single sniff. This significantly exceeds the detection capacity demonstrated by previous olfactory compressed sensing models. At the same time, our model is itself a recurrent circuit that demonstrates rich cell-type-specific neural dynamics in response to odor stimuli. These diverse dynamics qualitatively resemble those that have been measured in the mitral and tufted cells of the OB [21–25]. Thus, our work lays the groundwork for the development of performant circuit models that can generate detailed, experimentally-testable predictions about response dynamics in the olfactory bulb.

## 2 Simultaneous detection and estimation in olfactory scenes

Following the principle of analysis-by-synthesis, the starting point for our odorant recognition model is a generative model for olfactory scenes. At the most basic level, an odor landscape is determined by which sources are present; in the Montréal cityscape one might imagine coffee alongside bagels pulled freshly from a wood-fired oven. The presence of these sources then implies the presence of a characteristic set of volatile odorants which they emit. These odorants are then transported by turbulent airflow to the nose, where they can at last bind to olfactory receptors, driving activity in OSNs and thus generating a perceivable smell.

Importantly, there is a separation of the timescales at which odorant presence and measured concentration fluctuate. The underlying presence of an odorant varies relatively slowly, as sources appear and disappear. In contrast, the measured concentration at the olfactory sensory epithelium displays millisecond-timescale fluctuations over many orders of magnitude. These concentration fluctuations are driven both by the physical nature of turbulent transport in air before an odorant molecule arrives at the nose [14, 15, 48], and by the process of sniffing as a mammal actively inhales air into the nasal cavity [48]. This means that apparent concentrations of present odors will fluctuate wildly—and can indeed appear to be zero, as in some regimes of turbulence an odorant will appear only in whiffs [15]—but non-present odorants will remain absent until the sources change.

These physical and statistical distinctions suggest that it should be computationally advantageous to separately infer which odorants are present in a given scene, and the underlying concentration of the present odorants. This separation is reminiscent of the principles underlying simultaneous localization and mapping (SLAM) algorithms for visually-guided navigation in robots, which separate the inference of an agent’s rapidly-changing position from that of a map of slowly-changing landmarks [41]. From a probabilistic perspective, it corresponds to treating separately the distribution over presence p and the conditional distribution of concentration c given p:

(1) P(p,c)=P(c∣p)P(p).

Here, we focus on inference from a single, static snapshot of the olfactory world, modeling the decoding of odorant presence from a single sniff [13]. As we will contemplate at some length in the Discussion, this means that our inference model cannot take advantage of concentration measurements across multiple sniffs, which would allow it to directly leverage the separation of timescales in presence and concentration dynamics. However, focusing on static snapshots makes it easier to develop and test the formalism for olfactory SLAM we introduce here. This lays required groundwork for future incorporation of dynamical priors.

With this setup, we can now state the statistical model for odor scenes that we use in this work. We assume that there are $n_{\text{odor}}$ potential odorants, and $n_{\text{OSN}}$ types of olfactory sensory neurons, with $n_{\text{odor}}\gg n_{\text{OSN}}$. Thus, $c\in R_{+}^{n_{\text{odor}}}$, while $p\in \{0,\;1{\}}^{n_{\text{odor}}}$. We first specify a model for OSN firing rates as a function of apparent concentration, which gives the likelihood in our statistical model. Then, in keeping with the discussion above, we separately specify priors on the presence of odorants and of the concentration of present odorants. For simplicity, we assume that different odorants are independent and identically distributed under the prior. This could be relaxed, but as a starting point we focus on the i.i.d. case here (see Discussion).

To determine the likelihood, we model the mean activity of OSNs as a linear function of the concentration, with a receptor affinity matrix $A\in R_{+}^{n_{\text{OSN}}\times n_{\text{odor}}}$ and a baseline rate $r_{0}\in R_{+}^{n_{\text{OSN}}}$. This is an accurate model within moderate concentration ranges [49], but neglects nonlinear effects which are known to be important in olfactory sensing; see the Discussion for details. Following past works [29, 39], we then use a Poisson noise model for OSN activity $s\in R_{+}^{n_{\text{OSN}}}$. The OSNs fire independently, so the overall likelihood is $P(s|c,p)=\prod_{j=1}^{n_{\text{OSN}}}P\left(s_{j}|c,p\right)$ for

(2) $$P\left(s_{j}|c,p\right)=\text{Poisson}\left(r_{0}+\sum_{i=1}^{n_{\text{odor}}}A_{ji}p_{i}c_{i}\right),$$

where we assume that the OSNs have identical baseline firing rates $r_{0}$, and use in the likelihood the presence-masked concentration $p_{i}c_{i}$. We will compare several toy models for the distribution of affinities. In defining the affinity matrix A, we make the simplifying assumption that each OSN expresses a distinct receptor type. This disregards the fact that although each OSN expresses a single receptor type, there are multiple OSNs that express the same receptor type at different levels that converge on the same glomerulus [9, 16, 43, 44].

**Figure 2:**
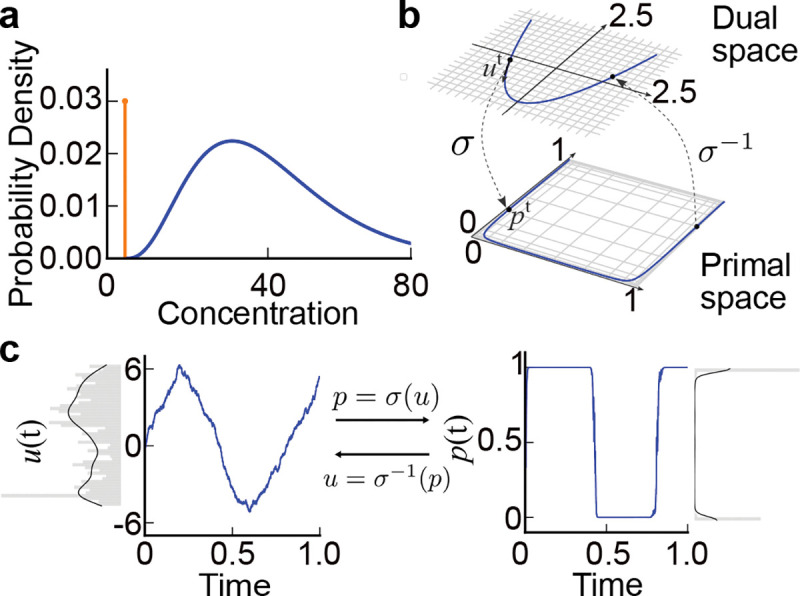
Prior and Mirrored Langevin dynamics. **a.** Spike and slab prior over concentration, which is a mixture of a Dirac delta function (orange curve) and a Gamma distribution (blue curve). **b.** Mirror map illustrating the nonlinear transformation between dual and primal spaces with the mirror map σ and its inverse $\sigma^{-1}$. Here, we set the gain of the sigmoidal mirror map σ to γ=3. This transformation projects a bounded interval of primal space to an unconstrained dual space. The sampling is performed in the dual space and projected back to the primal space. c. Example dynamics of the dual variable u(t) and the corresponding primal variable p(t). The mirror map σ is a sigmoid with gain γ=5. The distribution of samples in the primal space is concentrated near the boundaries at O and I.

We now specify a realistic ‘spike and slab’ prior on the presence-masked concentration $c_{i}p_{i}$, where the ‘spike’ assigns a large prior probability to the measured concentration being zero, and the ‘slab’ captures the broad distribution of concentrations of present odorants (Figure 2a). Using masking makes this easy, as by specifying a prior on presence that favors $p_{i}=0$ we immediately obtain the desired ‘spike’ at zero in the distribution of $p_{i}c_{i}$. For simplicity, we use a Bernoulli distribution

(3) $$P\left(p_{i}\right)=\varpi^{p_{i}}(1-\varpi {)}^{1-p_{i}}$$

for ϖ small. Thus, under the prior, the average number of present odors is $E\left[\sum_{i=1}^{n_{\text{odor}}}p_{i}\right]=n_{\text{odor}}\varpi$. Now we must specify the prior on c∣p. In the final estimated concentration $c_{i}p_{i}$, the value of $c_{i}$ is unconstrained when $p_{i}=0$. Thus, for simplicity we use the same prior as when $p_{i}=1$ (see Appendix D and Figure S3 for a discussion of presence-dependent priors). To mimic the broad distribution of odorant concentrations, we use as our prior on $c_{i}$ a Gamma distribution with parameters α and β:

(4) $$P\left(c_{i}|p_{i}\right)=Gamma\left(c_{i}|\alpha ,\beta \right).$$

This gives us the desired ‘slab’ for present odors (Figure 2a).

This probabilistic model for olfactory scenes was used in previous work by Grabska-Barwińska *et al*. [29, 50]. However, they did not provide a circuit implementation, limiting the ability of their model to generate testable predictions for experiment. Moreover, they did not show that their model could scale to scenes with more than a handful of odorants. In [39], we provided a circuit algorithm for non-separated inference of presence and concentration, but likewise found a limited capacity of only tens of odorants out of a repertoire of up to about 8000. It is these two limitations which we seek to overcome in this work.

## 3 Estimating presence through mirrored Langevin dynamics

The key obstacle to a recurrent circuit sampler is the fact that the presence is binary. This means that one cannot use the standard approach of writing down the Langevin dynamics that sample the posterior [29, 39, 51]. Grabska-Barwińska *et al*. [29] used Gibbs sampling to estimate odor presence, which maintains the exact binary nature of presence estimates, but cannot be readily mapped onto a rate-based recurrent neural network. To resolve this issue, we consider a continuous relaxation of the presence variables from {0, 1} to [0, 1]. Then, we can leverage the framework of Mirrored Langevin Dynamics (MLD), which allows Langevin sampling to be applied to constrained problems [42]. We emphasize that we will not endeavor to provide mathematically rigorous guarantees for the efficacy of our models—indeed, not all of the relevant distributions are log-concave, see Appendix B for details—and will rely on simulations.

Building on the framework of mirror descent [52, 53], the core idea of MLD is to express the constrained variable of interest in terms of an unconstrained “dual space” variable, and to sample using unconstrained Langevin dynamics in the dual space [42] (Figure 2b). Importantly, this mapping between the constrained “primal space” and the dual space is defined using a “mirror map”, so that it is reversible. This framework provides a powerful way to sample from constrained distributions using recurrent dynamics, but to our knowledge has not seen previous application in neuroscience. We therefore give a general, informal overview of the MLD framework in the Methods.

In the present case, we express $p\in [0,1{]}^{n_{\text{odor}}}$ in terms of a dual variable $u\in R^{n_{\text{odor}}}$:

(5) $$p_{i}=\sigma_{\gamma }\left(u_{i}\right),\;i\in \left[n_{odor}\right],$$

where the mirror map

(6) $$\sigma_{\gamma }(x)=1/\left(1+e^{-\gamma x}\right)$$

is the sigmoid with gain γ, and where we use the notation $\left[n_{\text{odor}}\right]=\left\{1,\ldots ,n_{\text{odor}}\right\}$. With this choice of mirror map, we can follow the MLD recipe to obtain a rate-based circuit that samples the posterior of interest. We give a detailed derivation in Appendix B, and state the final circuit model here. Let $h\in R^{n_{\text{OSN}}}$ be the ratio between the observed snapshot of OSN activity and the mean prediction from the likelihood based on the current concentration estimate $c_{i}(t)p_{i}(t)$, that is, a ratiometric prediction error [39]:

(7) $$h_{j}(t)=\frac{s_{j}(t)}{r_{0}+\sum_{i=1}^{n_{\text{odor}}}A_{ji}c_{i}(t)p_{i}(t)},\;j\in \left[n_{OSN}\right].$$

In terms of h(t), the dynamics of the dual presence variable u(t) and the latent concentration variable c(t) are:

(8) $$\begin{array}{l} du_{i}(t)\;=\frac{\gamma }{\tau_{u}}\left[p_{i}\left(1-p_{i}\right)\left(c_{i}\sum_{j=1}^{n_{OSN}}A_{ji}\left(h_{j}(t)-1\right)-\rho \right)-2p_{i}+1\right]dt+\sqrt{\frac{2}{\tau_{u}}}dB_{u,i}(t)\\ dc_{i}(t)\;=\frac{1}{\tau_{c}}\left[p_{i}\left(\sum_{j=1}^{n_{OSN}}A_{ji}\left(h_{j}(t)-1\right)+\frac{\alpha -1}{c_{i}}\right)-\beta \right]dt+\sqrt{\frac{2}{\tau_{c}}}dB_{c,i}(t) \end{array},i\in \left[n_{\mathrm{odor}}\right],$$

where $dB_{u}$ and $dB_{c}$ are independent $n_{\text{odor}}$-dimensional isotropic Gaussian noises, and the presence prior parameter is packaged into ρ=-log[ϖ/(1-ϖ)]. We define ρ such that it is positive when ϖ<1/2, *i.e*., when odors are more often not present under the prior. Here, we have also introduced time constants $\tau_{u}$ and $\tau_{c}$ to set the timescale of the inference dynamics; these should be similar to ensure reliable sampling.

Because this model performs simultaneous detection and estimation in olfactory scenes, we will refer to it by the acronym “SDEO”. If we simulate the dynamics (8) with modestly large gain values, the mirror map translates a relatively smoothly-varying dual space signal u(t) into a neatly nearly-binarized presence estimate p(t) (Figure 2c). Therefore, at the most basic level, the continuous relaxation yields an interpretable estimate of presence. The joint presence-concentration dynamics involve multiple forms of gating; the dynamics for u are in particular gated both by presence and concentration through $p_{i}\left(1-p_{i}\right)c_{i}$. If α>1, the divisive $1/c_{i}$ term from the concentration prior acts as a repulsive barrier preventing concentration estimates from going to zero.

**Figure 3:**
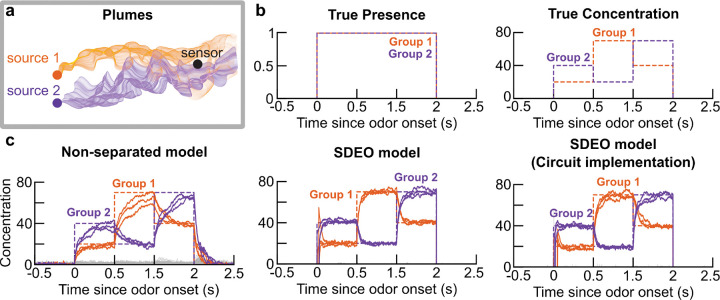
Dynamics of non-separated and SDEO models during estimation of odorants present in a slowly-changing scene. **a**. A static sensor downstream of two odor sources will measure an intermittent, variable mixture of the emitted odorants as they are transported by turbulent airflow. We illustrate this with a sketch inspired by the plume measurements of Nowotny and Szyszka [48]. **b**. In the laboratory, it is challenging to mimic the fast-timescale dynamics of a turbulent plume, but one can generate changing steps of concentration [14, 18, 19]. We model this scenario by selecting two groups of three random odorants each. Each group—intended to model the odor emitted by one source—has common concentration fluctuations, but remains present throughout the entire *in silico* experiment. Within each group, the concentrations of each odorant are identical, but the concentrations for the two groups change independently. We present this simulated odor scene to three models: non-separated (*left*; as in [39]), SDEO (*center*)), and SDEO with circuit implementation (*right*). In each plot, the colored lines denote the estimated concentration for the presented odorants, while the gray lines represent those for the background (non-presented) odorants. The dashed line traces true concentration over time. In these simulations, we use a sparse binary affinity matrix. For details of our numerical methods and the corresponding plot using a dense gamma-distributed affinity matrix, see Appendix F.4.2 and Figures S1 and S2.

As shown in Figure 3c, our SDEO model accurately tracks the presence and concentration of changing odorants in a simple scene. Here, we simply model the sensitivity matrix A as a sparse binary matrix; see Figure S2 for a similar test with a different model for the affinity matrix. Fixing p=1 and running Langevin sampling to infer c, we recover the model studied in our previous work [39] (Figure 3c). Returning to Figure 3 and comparing this non-separated model to the SDEO model developed above, we see that that the new model converges far more rapidly to a more accurate estimate of presence and concentration upon changes in the olfactory scene. This performance improvement—even in a relatively simple scene—is consistent with our conceptual motivations for considering split inference of presence and concentration.

## 4 Cell-type-specific computations and dynamics

To this point, we have shown how MLD allows us to build a recurrent circuit model for olfactory SLAM, and demonstrated that it can enable faster inference relative to an analogous model that simultaneously infers presence and concentration. We now seek a biological interpretation for this model, in particular one that attributes distinct functional roles to distinct cell types.

### 4.1 Removing divisive nonlinearities by introducing auxiliary neurons

**Figure 4:**
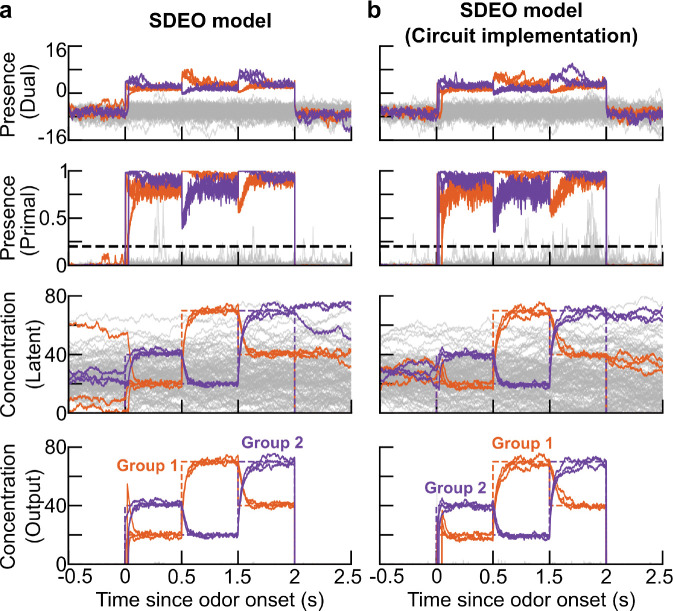
Internal dynamics of SDEO models during estimation of odorants present in the slowly-changing scene shown in Figure 3. In **a** and **b**, we show the basic SEDO model and its circuit implementation, respectively. The first row shows the estimated dual-space presence variable **u**, the second row shows the estimated presence in the [0, 1]-bounded primal space **p** (with a dashed line marking the threshold used to binarize presence during inference), the third row shows the latent concentration estimate **c** (which does not return to zero for non-present odors because of our choice of prior), and the fourth row shows the output concentration estimate $c_{i}p_{i}$. The panels in the fourth row are identical to the corresponding panels in Figure S2c. In each plot, the colored lines denote the estimated concentration for the presented odorants, while the gray lines represent those for the background (non-presented) odorants. Fluctuations visible in the primal space presence for non-present odors occur for non-present odors with low estimated concentrations, for which the presence can fluctuate without substantially changing the masked concentration $c_{i}p_{i}$. This is visible in the fact that the output concentrations for non-present odors remain near zero in the fourth row. In these simulations, we use a sparse binary affinity matrix. For details of our numerical methods and the corresponding plot using a dense gamma-distributed affinity matrix, see Appendix F.4.2 and Figures S1 and S2.

As in practice we see that the presence estimate p is essentially binary (Figure 4a), we hard gate it in the dynamics of c while keeping the continuous relaxation in the dynamics of u. We set ${\tilde{p}}_{i}=1$ when $p_{i}\geq p_{th}$ and ${\tilde{p}}_{i}=0$ when $p_{i}<p_{th}$, where $p_{th}>0$ is a given threshold parameter. Replacing p with $\tilde{p}$ in the dynamics of c while keeping the dynamics of u unchanged, we have the dynamics of the hard-gated version of the model:

(9) $$dc_{i}(t)=\frac{1}{\tau_{c}}\left[{\tilde{p}}_{i}\left(\sum_{j=1}^{n_{OSN}}A_{ji}\left({\tilde{h}}_{j}(t)\;-\;1\right)+\frac{\alpha -1}{c_{i}}\right)-\beta \right]dt+\sqrt{\frac{2}{\tau_{c}}}dB_{c,i}(t),\;i\in \left[n_{odor}\right]$$

where ${\tilde{h}}_{j}(t)=\frac{s_{j}}{r_{0}+\sum_{i=1}^{n_{\text{odor}}}A_{ji}c_{i}{\tilde{p}}_{i}}$.

These dynamics include divisive non-linearities, which make it hard to implement in a biologically plausible way [54]. Following our previous work [39], we linearize the dynamics using the method proposed in Chalk *et al*. [55]. Concretely, we introduce three additional cell types with rates $h\in R^{n_{\text{OSN}}},\tilde{h}\in R^{n_{\text{OSN}}}$ and $z\in R^{n_{\text{odor}}}$, with dynamics chosen such that they have as their fixed points the required divisions that define $h,\tilde{h}$, and the divisive prior term $(\alpha -1)/c_{i}=z_{i}$:

(10) $$\tau_{h}\frac{d}{dt}h_{j}\left(t\right)=s_{j}-h_{j}\left(r_{0}+\sum_{i=1}^{n_{\text{odor}\;}}A_{ji}c_{i}p_{i}\right),\;j\in \left[n_{OSN}\right],$$

(11) $$\tau_{\tilde{h}}\frac{d}{dt}{\tilde{h}}_{j}\left(t\right)=s_{j}-{\tilde{h}}_{j}\left(r_{0}+\sum_{i=1}^{n_{\text{odor}\;}}A_{ji}c_{i}{\tilde{p}}_{i}\right),\;j\in \left[n_{OSN}\right],$$

(12) $$\tau_{z}\frac{d}{dt}z_{i}\left(t\right)=\alpha -1-z_{i}c_{i},\;i\in \left[n_{odor}\right].$$

Substituting the instantaneous rates for the divisions in the dynamics for c and p, we obtain a circuit model with five cell types. If the time constants $\tau_{h},\tau_{\tilde{h}}$, and $\tau_{g}$ are small relative to the timescale of the dynamics of p and c, we expect this expanded circuit to perform comparably well to the model where the divisions are computed exactly [39, 55].

We can map these dynamics on the circuit architecture of the olfactory bulb [7, 56]. As they are excited by the OSN input, we interpret h and $\tilde{h}$ as the two classes of projection neurons in the OB (mitral and tufted cells). Then, the concentration estimate c and presence estimate p are encoded by local interneurons (granule cells), which inhibit the projection neurons and gate each other’s dynamics. The z neurons required to linearize the prior can then be interpreted as a form of cortical feedback onto the granule cells. With this coarse mapping in mind, we use time constants that are comparable to those measured in experiment for the major cell types of the olfactory bulb: we set $\tau_{h}=\tau_{\tilde{h}}=20\;ms$ to roughly match the primary projection neurons of the bulb, the mitral and tufted cells [57], and we set $\tau_{u}=\tau_{c}=30\;ms$ to roughly match the primary local interneurons of the bulb, the granule cells [58].

Though the resulting timescales are clearly not substantially separated, reliable inference is possible despite the resulting approximation to the divisions. In particular, the dynamics of this elaborated circuit model closely match that for the algorithmic split inference model introduced before (Figure 4a–b), with performance remaining far superior to the non-split model (Figure 3c). With this performance check in hand, we now turn to a more careful study of the dynamics of the different cell types in our model.

### 4.2 Dynamics and tuning of the hard- and soft-gated projection neurons

Although computational models of the olfactory bulb have provided great insights into the computational role of different cell types, they have usually ignored the differences between the two distinct dominant classes of projection neurons, the mitral and tufted cells [28, 29, 39, 50]—but see Tootoonian and Schaefer [59]. Suggestively, our elaborated model as introduced above has two classes of projection neurons—hard and soft threshold, respectively—which raises the question of how their responses compare to known properties of mitral and tufted cells.

**Figure 5:**
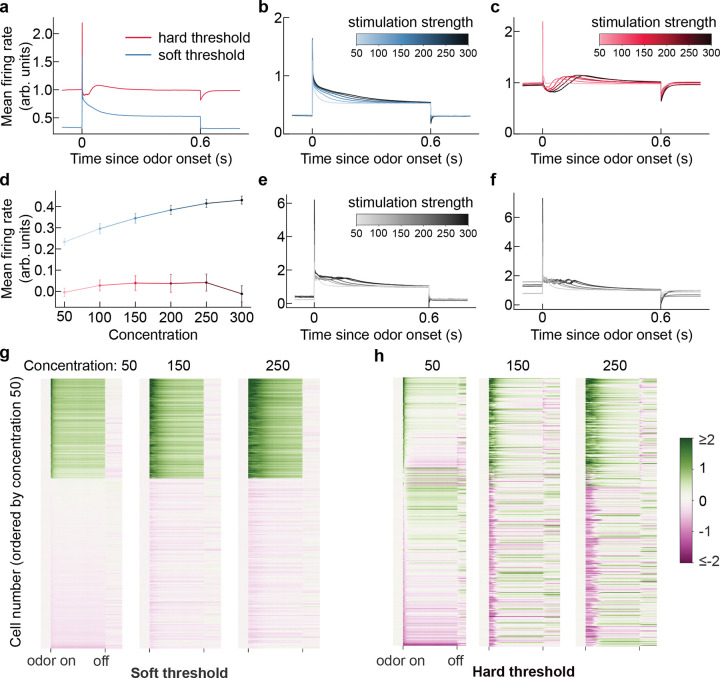
Comparison between hard gated projection neuron $\tilde{h}$ (labeled as hard threshold) and soft gated projection neuron h (labeled as soft threshold). A set of fixed odor stimuli is present at 0s and withdrawn at 0.6s. We set the Gamma distribution as the concentration prior with shape parameter α=5 and rate parameter β=0.1, and the Bernoulli prior for presence with ϖ=0.01. **a** Mean firing rate over time of two thresholding projection neurons. Upon stimulation, both the hard-gated neuron and the soft-gated neuron exhibit a transient burst of activity. After the burst, soft-gated neurons reduce to a plateau higher than baseline, while hard-gated neurons drop immediately, then rise again before settling into baseline activity. When the stimulation disappears, soft-gated neurons drop to baseline instantly, whereas hard-gated neurons exhibit a negative peak and then converge to baseline smoothly. **b.** Mean firing rate of soft-gated neurons under varying odor concentrations. Darker color indicates stronger stimulation. At odor onset, higher concentrations evoke longer-lasting responses, yet all activities converge to the same plateau. **c.** Mean firing rate of hard-gated neurons under varying odor concentrations. At odor onset, higher concentrations evoke deeper initial drops followed by a slower but higher rise. At odor offset, higher concentrations evoke lower negative peak, after which all responses converge back to baseline **d.** Mean firing rate as a function of odor concentration, averaged over the first 100 ms after odor onset. Error bars indicate standard error across trials. The soft-gated neurons respond monotonically to concentration. **e.** Example dynamics of a single soft-gated neuron under varying concentration. **f.** Example dynamics of a single hard-gated neuron under varying concentration. **g, h.** Baseline-subtracted activity of 300 soft-gated neurons and 300 hard-gated neurons, ordered by the response magnitude at concentration 50 (averaged over 50 ms after odor onset). A subset of soft-gated neurons and hard-gated neurons exhibits a stronger and loner peak at odor onset when concentration increases. The rest soft-gated neurons are stable, remain baseline or slightly below, whereas the remaining hard-gated neurons exhibit a negative peak, after which some recover to baseline and others rise above baseline. For additional single-neuron responses, see Figure S4.

In simple scenes modeling the static odorant mixtures presented in experiments, we find that our model recapitulates the differences in response amplitude, concentration dependence, and duration that are characteristic of mitral and tufted cells. At the population level, both classes exhibit a transient burst of activity at the time of odor onset (Figure 5). Soft-gated neurons then relax to a plateau above the baseline whose early amplitude and duration increase monotonically with concentration (Figure 5a,b,g). This is consistent with the response dynamics of the single class of projection neurons in our previous work [39] and with what has been observed experimentally in tufted cells [21, 22, 25]. By contrast, hard-gated neurons show a biphasic pattern: a steep suppression below baseline after the burst and then raising slowly before settling into baseline activity. Higher concentrations evoke deeper initial drops followed by a slower but slightly higher rise. (Figure 5a,c,h), consistent with the more complex dynamics of mitral cells [21, 22, 25]. Figure 5d further quantifies how stimulus intensity shapes the response dynamics by plotting the time-window–averaged firing rate (0–100 ms after onset) as a function of concentration, which suggests that soft-gated neurons (putative tufted cells) have a clearer monotonic response to increases in concentration that hard-gated neurons (putative mitral cells) as observed experimentally [25].

Examining baseline-subtracted single neuron responses, we can see that there are activated and suppressed neurons in both classes (Figure 5e–h). The activated soft-gated neurons exhibit sustained activities during stimulation. The suppressed soft-gated neurons are stable, remaining at or slightly below baseline. In contrast, activated hard-gated neurons show transient bursts, which are strengthened and prolonged as concentration increases. The other hard-gated neurons show a negative peak, after which followed by one of three outcomes: persistent suppression, return to baseline, or raise above baseline. These contrasting motifs identified here yield experimentally-testable predictions for the diversity of neural response dynamics.

However, the model’s response dynamics still deviate from experimental observations, particular in onset latency, sparsity, and the response threshold [21, 22, 25, 60]. We will return to this issue, and more broadly to the limitations of how well our model can be mapped to the circuitry of the olfactory bulb, in the Discussion. These limitations notwithstanding, these results illustrate the potential of our framework to generate testable predictions for cell-type-specific dynamics.

### 5 Scaling of olfactory inference

Thus far, we have shown that our model yields biologically-plausible computational dynamics. This fulfills one important desideratum of models for olfactory sensing. However, an algorithm must also be performant. Therefore, we developed two scaling simulations to evaluate our model under large network size and high dimensionality. The first quantifies the model’s ability to estimate odorants on a timescale of hundreds of milliseconds, while the second examines how the required number of OSNs scales with the size of potential odorant dictionary.

As a baseline, we use the non-split inference model we proposed in [39]. For the non-separated model which lacks explicit presence estimation, we approximate presence by binarizing the concentration estimates using half of the true concentration as the threshold. In general, to show the higher performance of the SDEO model, our evaluation criteria are much stricter than those used in [39]. We give a comprehensive discussion of our rationale for the choice of metrics in Appendix F.3.

### 5.1 Speed of inference

**Figure 6:**
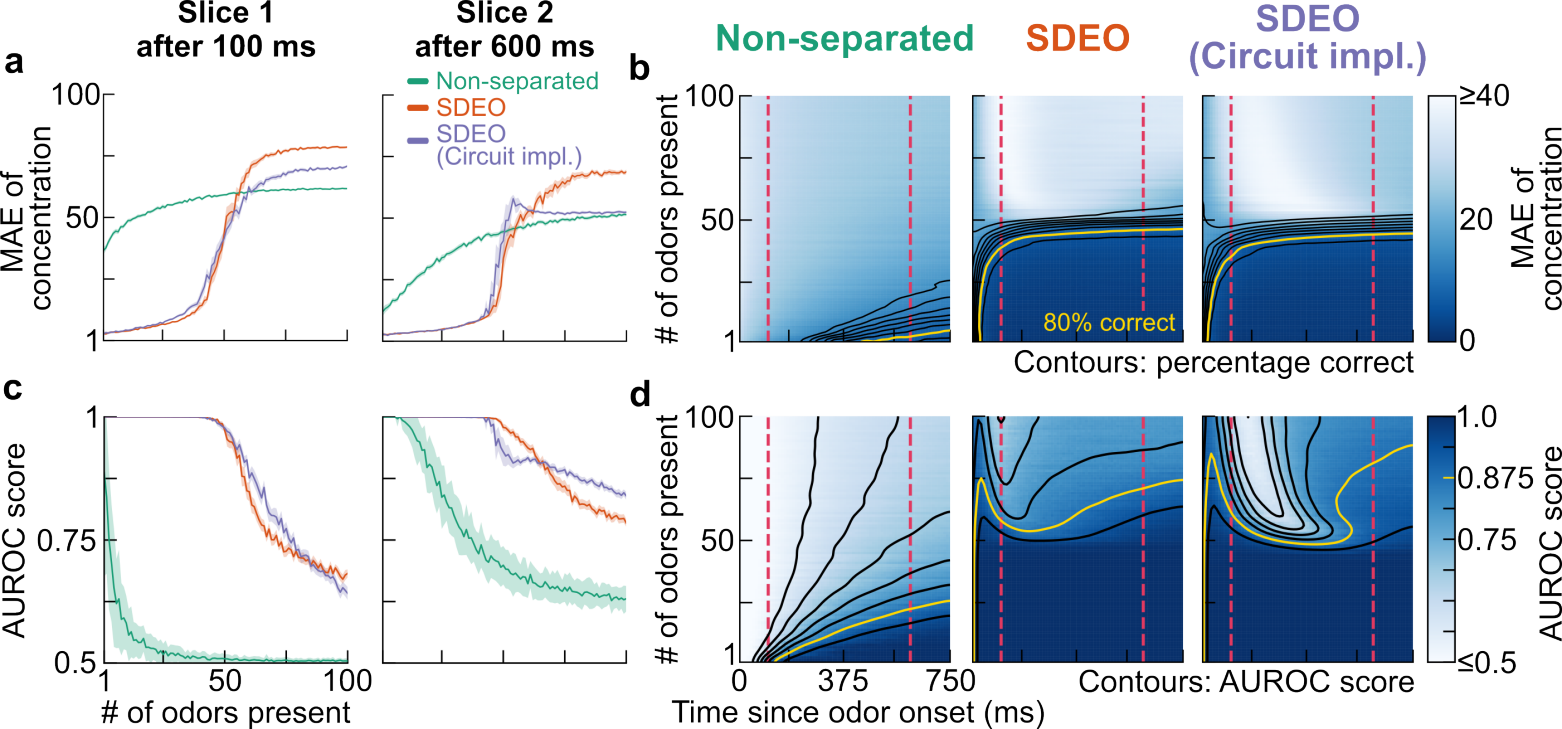
Improvement in fast detection of multiple odorants through separation of inference. We evaluate the same three models as in Figure 3 in a series of simulation where increasing numbers of odorants are simultaneously presented. In each run, in a set of 1000 odorants, a number of them are randomly selected and presented to the model for a duration of 0.75 s. We increase number of presented odors from 1 to 100, while repeat each setting for 40 times, compute the metrics and then take the average as final results. The shaded areas in **a** and **c** show ±1.96 · SEM (representing 95% C.I) over realizations throughout. The first and second rows assess the models’ performance in odorant concentration and presence estimation, respectively. **a.** Mean absolute error of estimated concentration as a function of the number of odorants present at two timepoints after odor onset. **b.** Heatmap of mean absolute error over inference time and number of presented odorants, with smoothed contours of correct detection fraction overlaid. **c.** AUROC score as a function of the number of odors present at two timepoints after odor onset. **d.** Heatmap of AUROC score over inference time and number of presented odors, with smoothed contours overlaid. For details of implementations and corresponding figures using dense Gamma sensing matrices, see Appendix F.4.3 and Figure S5.

We first evaluate our model’s ability to infer odor presence rapidly. With a potential odorant dictionary of size 1000 and 300 OSNs, the SDEO models can detect the concentration of ~40 simultaneously presented odorants with at least 80% accuracy and mean absolute error ≤ 25% · $c_{\text{True}}$ (Figure 6b,d; see also Figure S5). For presence estimates we can also see a similar but slightly improved limits of detection at ~50 simultaneously presented odorants. On the other hand, the non-separated model can only detect concentration of less than 10 odorants reliably, and detect presence reasonably accurate for at most 25 odorants. Moreover, we can see that SDEO models have significantly faster convergence rate where they reach steady states after ~100 milliseconds, while the non-separated model needs seconds to converge. In both concentration and presence estimation, the SDEO models demonstrate considerable improvement in both estimation speed and maximum number detection capacity compared to non-separated model under the same conditions.

Noticeably, we have also found the SDEO models exhibit a clear phase transition. Although having higher capacity compared to the non-separated model, the SDEO models collapse dramatically once the number of presented odorants exceeds their capacity, indicating qualitative changes in the network dynamics (Figure 6b,d; see also Figure S5). In contrast, the performance of the non-separated model deteriorates more gradually as the number of presented odorants increases. We also note that the phase transition for concentration and presence estimation occurs simultaneously at ~50 presented odorants. This reflects the strong correlation between concentration and presence estimation in the SDEO models and suggests that the models indeed leverage the coupled dynamics to achieve better sampling rather than merely carrying out two independent tasks.

### 5.2 Detection capacity

We now consider detection capacity, which we define as the detection capacity as the maximum number of simultaneously presented odorants that a model can detect with desired accuracy (see Appendix F.3.4 and the figure captions for details). From a compressed sensing perspective, the crucial question is how the number of sensors required to achieve a particular detection capacity scales with the size of the dictionary of potential odorants [26, 61].

**Figure 7:**
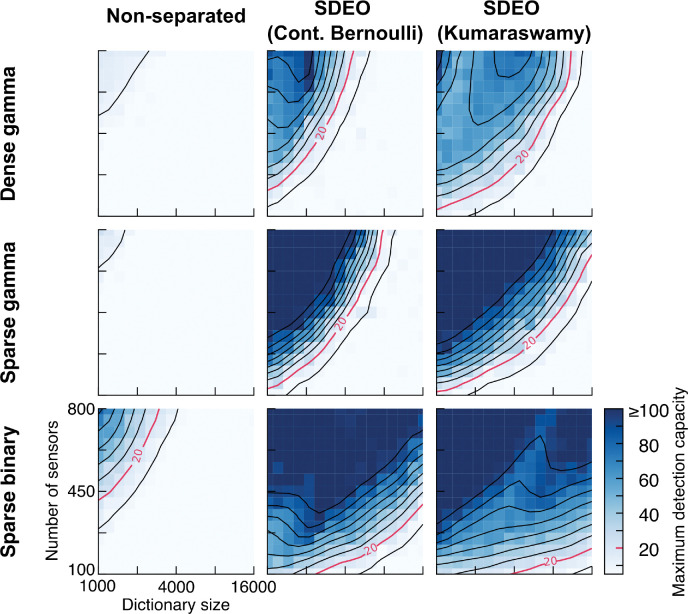
Scaling of detection capacity with dictionary size and sensor repertoire for different priors and sensing matrix models. The three columns correspond to three models—non-separated, SDEO and SDEO with circuit implementation—and the three rows correspond to three types of affinity matrices. These are: dense gamma, whose entries are i.i.d. random variable following Gamma(0.37, 0.36); sparse gamma, obtained by applying a 0.1 sparsity mask to a dense gamma matrix; and sparse binary, whose entries are i.i.d. random variable following Bernoulli(0.1). Each heatmap shows the maximum detection capacity assessed by presence estimates for combinations of sensors counts (from 100 to 800, equally spaced linearly) and dictionary size (1000 to 16000 equally spaced on a log scale). The maximum detection capacity $\kappa_{AUROC}$ is defined as the largest number of simultaneously presented number of odorants that the model can detect with a AUROC score ≥ 0.85. Smoothed contours are overlaid and can be interpreted as the required number of sensors to maintain a certain capacity as a function of dictionary size. The total inference time duration is 0.2 s for all runs, and the value in each cell of the heatmap is the average of three independent runs. For further discussion on affinity matrix and scaling capacity, see Appendix E. For additional details of implementation and the corresponding figures using mean absolute error to compute maximum detection capacity, see Appendix F.4.4 and Figure S6.

In Figures 7 and S6, we examine the scaling of detection capacity with dictionary size and sensor repertoire across different combinations of models and sensing matrix. In the first two columns, we can see the SDEO model significantly out-performs the non-separated model in the range of entire heatmap. Thus, splitting the inference of presence and concentration improves the speed and accuracy of inference, yielding higher capacity. Upon closer inspection of the SDEO model’s dynamics, we find that the ultimate bottleneck of capacity arises from presence estimation, which drives the sampling of concentration.

### 5.3 Improving capacity by modifying the presence prior

As detection capacity appeared to be bottlenecked by presence estimation, we therefore sought to improve presence estimation by modifying the corresponding prior. Up to this point, we have treated the presence of odorants as a binary variable, and correspondingly specified for it a Bernoulli prior. However, this is an imperfect choice under the continuous relaxation for presence estimation. The natural distribution of presence probability is bimodal on [0, 1] with density clustering near the endpoints. However, the density of the continuous Bernoulli prior decreases monotonically on [0, 1] with our chosen parameter (Figure S7). To overcome this issue in the continuous relaxation of presence estimation, we want a naturalistic bimodal distribution on [0, 1] that can capture the natural distribution of presence probability. Ideally, such a distribution should also admit a tractable density and score.

The obvious candidate for such a prior would be the Beta distribution, but its density and score are non-trivial to analyze and costly to compute. Past works have proposed the use of the Kumaraswamy (KS) distribution as an alternative to the Beta distribution in similar contexts [62–64]. Unlike the Beta distribution, the KS distribution involves solely elementary functions, and is therefore straightforward to incorporate into our model. However, the vanilla KS distribution suffers from instability issues due to exploding gradients near boundaries of its open support (0, 1). We overcome this by mapping a truncated KS distribution on the closed interval [0+ε,1-ε] to the full interval [0, 1] for a small cutoff $\varepsilon ∼O\left(10^{-5}\right)$ (see Appendix C for details). This truncated KS prior allows for efficient and numerically-stable computation.

As shown in Figures 7 and S6, the SDEO model with truncated Kumaraswamy distribution prior on presence empirically outperforms the model with a continuous Bernoulli prior. Given the same odorant dictionary size, fewer sensors are required to reach a given capacity. Moreover, the limiting dimensionality is also increased, depicted by delayed explosion in the slope of the contours. This improvement is perhaps because the bimodal Kumaraswamy distribution better accounts for the underlying structure of presence, thus facilitating efficient sampling.

Just as altering the prior improves the capacity, similar improvements result from sparsifying the affinity matrix: dense Gamma-distributed affinity matrices yield the lowest capacity, followed by sparse Gamma, and then by sparse binary. As we analyze and discuss in Appendix E and Figure S8, this improvement likely results from the decreased mutual coherence—the maximum overlap between the affinity profiles of two sensors—of the sparse ensembles relative to the dense one, consistent with the existing compressed sensing literature [61].

In sum, particularly with an appropriate choice of prior and of affinity matrix, our SDEO model can detect and estimate the concentration of up to a hundred or so odorants out of a repertoire of tens of thousands (Figures 7 and S6). This significantly exceeds the capacity of the model studied in our previous work, and of other models for olfactory compressed sensing.

## 6 Discussion

In this work, we have combined insights from simultaneous localization and mapping in robotics [41], from compressed sensing [26], and from mirrored Langevin dynamics sampling algorithms [42] to propose a biologically-constrained model for odorant sensing (Figure 1–3). Our algorithm recapitulates the experimentally observed distinct properties of the two main projection neuron classes in the bulb, mitral and tufted cells (Figure 5). We further show that our approach scales to large sensory scenes, successfully detecting the presence and estimating the concentration of tens to hundreds of odorants amongst thousands of potential ones (Figure 6–7).

Our model suggests a map between anatomy and computational function, with the tufted cells contributing to the odor presence detection and the mitral cells to the concentration estimation computation. The dynamics of each cell type is broadly consistent with experimental observations [21, 22, 25] (Figure 5) but our model leaves open how the information is used by cortical areas. The major cortical projection targets are distinct across the two cell types—with, roughly speaking, tufted cells projecting predominantly to anterior cortical regions like the AON and mitral cells projecting to more posterior regions like the piriform cortex—but there are secondary projections with substantial projection from mitral cells into the AON and some projection from tufted cells into piriform cortex [22, 46, 47]. This implies that the computational function of cortical areas is not readily mapped onto the presence and concentration axes. Representations in piriform cortex have been shown to carry both concentration and presence information [65, 66], consistent with inputs from both mitral and tufted cells. Piriform also carries contextual information such as location [67], which could play a role in adapting the odor-processing prior [68, 69]. Less is known about tuning to concentration in the AON but experimental data is consistent with a role in detection of odor identity which would be mediated by the tufted cell inputs in our model [70–73].

The circuit implementation of our model could also be extended by introducing a more detailed model of the granule cells and other cell types of the bulb [39, 74]. In contrast to previous accounts of the M/T computational split [59], our model proposes that these two cell types interact through indirect, reciprocal multiplicative gating rather than additive excitation. This gating effect could be probed in future experiments. As in our previous work [39], the M/T cells encode a ratiometric prediction error between the current OSN activity and the prediction thereof based on the current estimate of concentration encoded in the granule cells. However, by introducing hard and soft gating, we form two predictions, and correspondingly two types of prediction error neurons. Existing models for predictive coding in other sensory systems that incorporate multiple cell types generally assume Gaussian noise models, and correspondingly subtractive prediction errors [75]. It will be interesting to contrast predictions of models encoding subtractive versus ratiometric errors in future work.

Attempts to experimentally probe cell-type-specific response dynamics in the OB have traditionally been limited by the challenges inherent in controlling an odor stimulus. Unlike for vision or audition, it is challenging to deliver tunable olfactory stimuli that mimic the statistics of those encountered in the natural world. Typical experiments in psychology and neuroscience have delivered static odor flows at high concentrations over timescales of hundreds of milliseconds. It is only recently that experimental paradigms have allowed to combine recordings of neural activity with rapid control of the physical stimulus, through rapidly switching air flows for odor delivery [14, 18, 76, 77], invasive approaches controlling breathing [49], or direct optogenetic activation of olfactory sensory neurons [78–81]. Thus, it is now possible to test predictions from a model like ours about the neural dynamics in response to temporally-structured stimuli.

Despite the richness of its predictions, our model only provides at best a partial picture of the computational architecture of the olfactory bulb. As mentioned before, our work does not take full advantage of the olfactory SLAM framework, as we do not model the dynamics of the odor environment. A more complete circuit would incorporate into its prior a motion model for the slow dynamics of odor presence, as well as a prior for fast fluctuations in concentration due to sniffing [18] and turbulent transport [15, 48]. Here, we have neglected odor dynamics to focus on the minimal setting in which we can test implementations of olfactory SLAM using MLD. However, dynamical priors could be incorporated into this framework without much difficulty. This would in turn allow us to make richer predictions about cell-type-specific dynamics in the olfactory system [21, 24, 55], and improve inference from multiple samples of more complex scenes.

With the fact that one would eventually like to incorporate different dynamical priors on presence and concentration in mind, it is important to note that splitting the inference is not the only way to implement inference using a *static* spike-and-slab prior in a rate network. In recent work, Fang *et al.* [82] have proposed a Langevin sampling network for a spike-and-slab prior based on thresholding. As we detail in Appendix G, applying this methodology to the olfactory sensing problem leads to a gated RNN with a single population of neurons that directly encodes the sparse vector of concentration elements. It is not immediately clear how one could implement priors over concentration and presence with distinct timescales within this framework. Moreover, it cannot instantiate the separation of computational labor that is at the heart of the SLAM approach.

Additionally, we used a simplified model for OSN responses that assumes that receptor affinities are independent and identically distributed (iid), and that the mean firing rate of each OSN is linear in concentration. The assumption of iid receptor affinities makes it easier to systematically probe scaling properties of the model, but is an idealization. Using these iid affinities, we saw that improved scaling capacity was obtained for sparse random affinities, which as mentioned before is qualitatively consistent with ideas from the theory of compressed sensing with Gaussian noise. It is also consistent with previous works that have shown that optimizing affinities to maximize the mutual information between odor concentrations and OSN activities (assuming either linear or simple non-linear models for the firing rate, and Gaussian noise) leads to sparse affinity matrices [3, 4, 32]. In these optimized models, the non-zero affinities are broadly-distributed—which our sparse Gamma model aims to roughly capture—but the distributions of individual elements contain structure beyond long tails [4]. Investigating how our results change given optimized sensitivity matrices and non-linear OSNs could be an interesting avenue for future investigation, but the optimization procedures used in past works are too computationally expensive to enable the investigations of scaling we perform here.

Another level of structure which we have not yet incorporated into our model is correlations between the presence of different odorants that arise from their generation by a common source. Recent work has shown that such environmental correlations can shape the distribution of optimal receptor affinities [4]. Closer to our work here, Tootoonian and Schaefer [40] have recently proposed a circuit basis for the encoding of correlated odorant priors in the olfactory bulb. Their construction leverages sister mitral cells, which receive input from the same receptors but are connected to different granule cells [78]. Our model does not leverage this structure, and incorporating structure and hierarchies into the prior will be important as we move towards testing its ability to parse realistically-structured scenes.

Another substantial limitation of the present work is that we consider a non-distributed coding scheme, in which each granule cell represents the estimate of the presence or concentration of a specific odorant. This appears inconsistent with physiology [7, 70]. It also stands in contrast to our previous work on the non-split version of this inference model in [39]. There we showed that linearly distributing the odorant code—*i.e.*, writing the estimated concentration vector as c=Γg for model granule cell activities g and a decoding matrix Γ—allowed for significant improvements in performance if one chose Γ to cancel correlations in OSN responses induced by a dense sensing matrix. It is therefore possible that taking advantage of this geometry would close some of the gaps between different sensing matrix ensembles which we observed in Figure 7. However, the simple linear mixing we used in [39] will not suffice here, as the additional nonlinearities in the model dynamics fix a preferred basis. Naïvely following the linear mixing approach leads to a model without a clear circuit interpretation. Determining how to construct a split inference model with fully distributed coding will be an important task for future work. This will also be an important prerequisite to more direct comparison of cell-type-specific dynamics in models with those measured in the OB.

More generally, our work points to compositionality of neural representations, as has been studied across other modalities and neural circuits [83–85]. Decoupling concentration estimating and odor detection, would allow extensions of our model to learn complex priors over these two domains separately. For example the dependence on context of the structure of odor presence and of the dynamics of concentration fluctuations due to air flow to are likely to be only loosely correlated (presence of odor emitting objects vs properties of air flow in a given environment).

In closing, we emphasize that the framework we develop here can be adapted to a broad array of inference problems that arise in other sensory modalities, beyond olfaction alone. In particular, MLD provides a general recipe for the design of recurrent circuits that solve constrained probabilistic inference problems. Recent work has applied mirror descent—which is to optimization as MLD is to sampling—to study learning in the presence of biological constraints on synaptic weights [86]. However, the application of this toolkit to neural dynamics is to our knowledge novel. At the same time, MLD has recently gained popularity in machine learning as a method to enable learning how to sample from constrained distributions using diffusion generative models [87, 88]. It therefore holds much promise as a foundation for building circuit models that can process naturalistic, complex stimuli.

## 7 Methods

### 7.1 The Mirrored Langevin Dynamics recipe

Here, we give a brief introduction to the Mirrored Langevin Dynamics (MLD) framework introduced by Hsieh *et al.* [42], and summarize its application to presence estimation. Abstractly, suppose that we wanted to sample a distribution P(x) over a variable x that is subject to some constraint. MLD prescribes that we should define a convex function ϕ(x) that transforms the constrained variable x into an unconstrained dual-space variable y via the “mirror map”

(13) $$y=\nabla_{x}\phi \left(x\right),$$

and maps the un-constrained variable y back to the constrained variable x via

(14) $$x=\nabla_{y}\phi^{*}\left(y\right).$$

Here,

(15) $$\phi^{*}(y)=\underset{x}{sup}\;\{\langle y,x\rangle -\phi (x)\}$$

is the Fenchel dual—that is, the Legendre transform—of the convex function ϕ. With appropriate choices of mirror map, this framework can handle many classes of constraints, far beyond the simple hypercube constraint considered here [42].

Then, since y(t) is unconstrained, we can run naïve Langevin dynamics on y,

(16) $$dy\left(t\right)=\nabla_{y}\log Q\left(y\right)dt+\sqrt{2}dB_{u}\left(t\right),$$

for Gaussian noise $dB_{u}(t)$, where the stationary distribution Q(y) is chosen such that $x(y)=\nabla_{y}\phi^{*}(y)$ has the desired distribution P(x). For x(y) to have density P(x), the density of y should be

(17) $$Q\left(y\right)=det{\left(\nabla_{x}^{2}\phi \left(x\right)\right)}^{-1}P\left(x\left(y\right)\right),$$

as the definition of the Fenchel conjugate implies that the Jacobian of the change-of-variables $x(y)=\nabla_{y}\phi^{*}(y)$ is just inverse of the the Hessian of ϕ:

(18) $$\nabla_{y}x\left(y\right)=\nabla_{y}^{2}\phi^{*}\left(y\right)={\left[\nabla_{x}^{2}\phi \left(x\right)\right]}^{-1}\equiv H^{-1},$$

where we abbreviate the Hessian as $H\equiv \nabla_{x}^{2}\phi (x)$. By the chain rule, the score appearing in the Langevin dynamics for y is then

(19) $$\nabla_{y}\log Q\left(y\right)=H^{-1}\left[\nabla_{x}\log P\left(x\right)-\nabla_{x}\log detH\right],$$

where the right-hand-side of the equation should be viewed as a function of y through x(y). This informal sketch recovers the MLD prescription of Hsieh *et al*. [42].

For the case of presence estimation, where the constraint is just that p should lie within the hypercube $[0,\;1{]}^{n_{\text{odor}}}$, we can apply this recipe with

(20) $$\phi \left(p\right)=\frac{1}{\gamma }\sum_{j=1}^{n_{\text{odor}}}\left[p_{j}\log p_{j}+\left(1-p_{j}\right)\log\left(1-p_{j}\right)\right],$$

for any γ>0, which has Fenchel dual

(21) $$\phi^{*}\left(u\right)=\frac{1}{\gamma }\sum_{j=1}^{n_{\text{odor}}}\log\left[1+\exp\left(\gamma u_{j}\right)\right],$$

and thus yields as the mirror map the desired sigmoid

(22) $$\frac{\partial \phi^{*}}{\partial p_{j}}=\sigma_{\gamma }\left(p_{j}\right)=\frac{1}{1\;+\;e^{-\gamma p_{j}}}.$$

With this choice, for the SDEO posterior the MLD recipe as sketched above leads to the dynamics (8); we defer the full details of this derivation to Appendix B.

### 7.2 Summary of numerical methods

We now briefly summarize our numerical methods, deferring a detailed description to Appendix F. Our model is defined as a system of stochastic differential equations, which we integrate numerically using the Euler-Maruyama method with a fixed timestep of $\Delta t=10^{-5}$ seconds. To enable efficient simulation in high dimensions—as is required to study capacity scaling in Figure 7—we implement our simulations in PyTorch [89], which allows efficient, parallelizable multiplication of large matrices.

We evaluate concentration estimation using the mean absolute error between estimated and true concentrations of present odorants. As a complementary measure, we consider the proportion of correct concentration estimates, which we define as those for which the estimated concentration is within a ±25% interval of the true value. To assess the quality of presence estimation, we adopt the AUROC score, because inferring presence can be framed as a binary classification problem. For the non-separated model which lacks explicit presence estimation, we approximate presence by binarizing the concentration estimates using half of the true concentration as the threshold. We give a comprehensive discussion of our rationale for the choice of metrics in Appendix F.3.

## Supplementary Material

Supplement 1

## Data Availability

Code to reproduce all figures is available on GitHub at: https://github.com/labmasset/Simultaneous-detection-and-estimation-in-olfactory-sensing.
