# Supplementary material for: Simultaneous detection and estimation in olfactory sensing
Source: bioRxiv. 2025 Nov 3:2025.11.01.686013. Preprint. [Version 1] doi: 10.1101/2025.11.01.686013 (PMC12637662; doi:10.1101/2025.11.01.686013)
Supplement: Supplement 1 [file NIHPP2025.11.01.686013v1-supplement-1.pdf]

bioRxiv preprint doi: https://doi.org/10.1101/2025.11.01.686013; this version posted November 3, 2025. The copyright holder for this preprint (which was not certified by peer review) is the author/funder, who has granted bioRxiv a license to display the preprint in perpetuity. It is made available under aCC-BY 4.0 International license.

Supplemental Information

A Notational conventions

Table 1: Variable and parameter names

Variable name	Description	Type / Domain
**Dimensions**		
n_{odor}	Number of potential odorants (Dictionary size)	$\mathbb{N}$
n_{OSN}	Number of sensors (Sensor repertoire size)	$\mathbb{N}$
n_P	Number of odorants present	$\mathbb{N}$
**Variables**		
$\mathbf{s}$	OSN responses	$\mathbb{R}_+^{n_{\text{OSN}}}$
$\mathbf{h}$	Responses of soft-gated projection neurons	$\mathbb{R}_+^{n_{\text{OSN}}}$
$\tilde{\mathbf{h}}$	Responses of hard-gated projection neurons	$\mathbb{R}_+^{n_{\text{OSN}}}$
$\mathbf{u}$	Estimated presence in the dual space for all potential odorants	$\mathbb{R}^{n_{\text{odor}}}$
$\mathbf{p}$	Estimated presence in the primal space for all potential odorants	$[0,1]^{n_{\text{odor}}}$
$\mathbf{c}$	Estimated concentration for all potential odorants	$\mathbb{R}_+^{n_{\text{odor}}}$
**Single-neuron parameters**		
$\mathbf{r}_0$	Baseline firing rate of OSN	$\mathbb{R}_+^{n_{\text{OSN}}}$
**Population parameters**		
$\mathbf{A}$	Affinity matrix	$\mathbb{R}_+^{n_{\text{OSN}} \times n_{\text{odor}}}$
**Notation**		
$\odot$	Element-wise (Hadamard) vector product	Notation
$\oslash$	Element-wise (Hadamard) vector division	Notation
$\mathbf{B}$	Brownian noise (Langevin)	Notation
T	Time available for inference	Notation
$\mathbf{1}_{\{\cdot\}}$	Indicator function	Notation

B Derivation of the full model

In this Appendix, we give a detailed derivation of the inference model introduced in the main text. Before launching into the derivation, we note that we will use vector notation, with $\odot$ representing elementwise multiplication and $\oslash$ representing element-wise division, *i.e.*, for two vectors $\mathbf{a}$ and $\mathbf{b}$ we write $(\mathbf{a} \odot \mathbf{b})_i = a_i b_i$ and $(\mathbf{a} \oslash \mathbf{b})_i = a_i / b_i$.

We recall that our goal is to sample the Bayes posterior

$$P(\mathbf{c}, \mathbf{p} \mid \mathbf{s}) = \frac{P(\mathbf{s} \mid \mathbf{c}, \mathbf{p})\ P(\mathbf{c} \mid \mathbf{p})\ P(\mathbf{p})}{P(\mathbf{s})} \tag{B.1}$$

over odor presence $\mathbf{p} \in \{0,1\}^{n_{\text{odor}}}$ and concentration $\mathbf{c} \in \mathbb{R}_+^{n_{\text{odor}}}$ given a snapshot $\mathbf{s} \in \mathbb{R}_+^{n_{\text{OSN}}}$ of olfactory sensory neuron (OSN) activity. Following the simultaneous localization and mapping (SLAM) idea, we first specify a prior $P(\mathbf{p})$ on presence, and then a prior $P(\mathbf{c} \mid \mathbf{p})$ on the concentration of the present odorants.

bioRxiv preprint doi: https://doi.org/10.1101/2025.11.01.686013; this version posted November 3, 2025. The copyright holder for this preprint (which was not certified by peer review) is the author/funder, who has granted bioRxiv a license to display the preprint in perpetuity. It is made available under aCC-BY 4.0 International license.

As introduced in the main text, we aim to approximately sample the posterior by applying the framework of Mirrored Langevin Dynamics (MLD) [42] to a continuous relaxation of the presence variable to $[0, 1]^{n_{\text{odor}}}$. The posterior enters these dynamics only through the gradients of the potential

$$V = -\log P(\mathbf{c}, \mathbf{p} \mid \mathbf{s}) \tag{B.2}$$

with respect to $\mathbf{p}$ and $\mathbf{c}$, meaning that the marginal likelihood $P(\mathbf{s})$ does not enter the subsequent calculations. Throughout, we use a Poisson likelihood

$$P(\mathbf{s} \mid \mathbf{c}, \mathbf{p}) = \text{Poisson}(\mathbf{r}_0 + \mathbf{A}(\mathbf{c} \odot \mathbf{p})). \tag{B.3}$$

as in prior works [29, 39]. We leave the priors unspecified for now, as we will consider several different choices.

B.1 Deriving the Mirrored Langevin Dynamics for presence estimation

We now give a detailed derivation of the dynamics that result from applying the MLD recipe to joint inference of presence and concentration. As mentioned in the main text, we consider the mirror map

$$\mathbf{p} = \sigma_\gamma(\mathbf{u}) \tag{B.4}$$

where for any $\gamma > 0$,

$$\sigma_\gamma(x) = \frac{1}{1 + \exp(-\gamma x)} \tag{B.5}$$

is the logistic sigmoid with gain γ, taken to act elementwise. The gain factor γ allows us to control the steepness of the sigmoid. To apply the MLD recipe, our goal is to construct a convex function $\phi : [0, 1]^{n_{\text{odor}}} \to \mathbb{R}^{n_{\text{odor}}}$ with Fenchel conjugate $\phi^* : \mathbb{R}^{n_{\text{odor}}} \to [0, 1]^{n_{\text{odor}}}$ such that

$$\frac{\partial \phi^*}{\partial u_j} = \sigma_\gamma(u_j). \tag{B.6}$$

which means that we should have

$$\frac{\partial \phi}{\partial p_j} = \sigma_\gamma^{-1}(p_j) = \frac{1}{\gamma} \log \frac{p_j}{1 - p_j}. \tag{B.7}$$

The natural choice—as discussed by Hsieh *et al.* [42]—is then of course to take

$$\phi(\mathbf{p}) = \frac{1}{\gamma} \sum_{j=1}^{n_{\text{odor}}} [p_j \log p_j + (1 - p_j) \log(1 - p_j)], \tag{B.8}$$

which has Fenchel conjugate

$$\phi^*(\mathbf{u}) = \frac{1}{\gamma} \sum_{j=1}^{n_{\text{odor}}} \log[1 + \exp(\gamma u_j)]. \tag{B.9}$$

Here, we use the usual information-theoretic convention that $0 \log 0 = 0$.

As ϕ is additively separable, its Hessian is diagonal:

$$\frac{\partial^2 \phi}{\partial p_i \partial p_j} = \frac{1}{\gamma} \delta_{ij} \frac{1}{p_j(1 - p_j)}. \tag{B.10}$$

This allows us to easily obtain

$$\log\det \nabla^2 \phi(\mathbf{p}) = \sum_{j=1}^{n_{\text{odor}}} \log \frac{1}{p_j(1 - p_j)} - n_{\text{odor}} \log \gamma, \tag{B.11}$$

hence

$$\frac{\partial}{\partial p_j} \log\det \nabla^2 \phi(\mathbf{p}) = \frac{2p_j - 1}{p_j(1 - p_j)}. \tag{B.12}$$

bioRxiv preprint doi: https://doi.org/10.1101/2025.11.01.686013; this version posted November 3, 2025. The copyright holder for this preprint (which was not certified by peer review) is the author/funder, who has granted bioRxiv a license to display the preprint in perpetuity. It is made available under aCC-BY 4.0 International license.

For a potential $V = -\log P(\mathbf{p}, \mathbf{c} \mid \mathbf{s})$, the MLD recipe [42] then gives the dynamics

$$d\mathbf{u}(t) = -\boldsymbol{\nabla}^2\phi(\mathbf{p})^{-1}\left[\boldsymbol{\nabla}_{\mathbf{p}}V + \boldsymbol{\nabla}\log\det\boldsymbol{\nabla}^2\phi(\mathbf{p})\right]\,dt + \sqrt{2}d\mathbf{B}(t) \tag{B.13}$$

$$\mathbf{p}(t) = \boldsymbol{\nabla}\phi^*(\mathbf{u}(t)) \tag{B.14}$$

Substituting in the properties of our choice of ϕ, we have

$$du_j(t) = -\gamma\left[p_j(1-p_j)\frac{\partial V}{\partial p_j} + 2p_j - 1\right]\,dt + \sqrt{2}dB_j(t) \tag{B.15}$$

or, in vector form

$$d\mathbf{u}(t) = -\gamma\left[\mathbf{p}\odot(\mathbf{1}-\mathbf{p})\odot\boldsymbol{\nabla}_{\mathbf{p}}V + 2\mathbf{p} - \mathbf{1}\right]\,dt + \sqrt{2}d\mathbf{B}_u(t) \tag{B.16}$$

We can then jointly sample $\mathbf{c}$ and $\mathbf{u}$ by simultaneously running the dynamics

$$d\mathbf{c}(t) = -\boldsymbol{\nabla}_{\mathbf{c}}V\,dt + \sqrt{2}d\mathbf{B}_c(t) \tag{B.17}$$

for an independent Brownian motion $\mathbf{B}_c$. To be extremely formal, we can also introduce a trivial mirror map for $\mathbf{c}$, and consider the combined mirror map induced by

$$\phi(\mathbf{p}, \mathbf{c}) = \frac{1}{\gamma}\sum_j[p_j\log p_j + (1-p_j)\log(1-p_j)] + \frac{1}{2}\|\mathbf{c}\|_2^2, \tag{B.18}$$

which handles the constraint on $\mathbf{p}$ while leaving $\mathbf{c}$ intact.

We remark in passing that not all of the distributions of interest are log-concave in the dual space (which is the relevant notion for MLD [42]). Guarantees for the convergence of stochastic gradient Langevin dynamics given certain sufficient conditions when sampling on non-log-concave target distributions are weaker in general than those known in the log-concave case, but theoretical progress has in recent years been rapid [90, 91]. Since we are anyway relying on numerics rather than rigorous proofs for fast convergence, we will not dwell on this issue further.

B.2 Gradients of the combined presence-concentration posterior

We now explicitly compute the energy gradients that appear in the MLD. From the definition of the posterior (B.1), we have:

$$-V(\mathbf{c}, \mathbf{p}) = \log P(\mathbf{c}, \mathbf{p} \mid \mathbf{s}) = \log P(\mathbf{s} \mid \mathbf{c}, \mathbf{p}) + \log P(\mathbf{c} \mid \mathbf{p}) + \log P(\mathbf{p}) - \log P(\mathbf{s}), \tag{B.19}$$

so the required gradients are

$$-\boldsymbol{\nabla}_{\mathbf{c}}V(\mathbf{c}, \mathbf{p}) = \boldsymbol{\nabla}_{\mathbf{c}}\log P(\mathbf{c}, \mathbf{p} \mid \mathbf{s}) = \boldsymbol{\nabla}_{\mathbf{c}}\log P(\mathbf{s} \mid \mathbf{c}, \mathbf{p}) + \boldsymbol{\nabla}_{\mathbf{c}}\log P(\mathbf{c} \mid \mathbf{p}) \tag{B.20}$$

$$-\boldsymbol{\nabla}_{\mathbf{p}}V(\mathbf{c}, \mathbf{p}) = \boldsymbol{\nabla}_{\mathbf{p}}\log P(\mathbf{c}, \mathbf{p} \mid \mathbf{s}) = \boldsymbol{\nabla}_{\mathbf{p}}\log P(\mathbf{s} \mid \mathbf{c}, \mathbf{p}) + \boldsymbol{\nabla}_{\mathbf{p}}\log P(\mathbf{c} \mid \mathbf{p}) + \boldsymbol{\nabla}_{\mathbf{p}}\log P(\mathbf{p}) \tag{B.21}$$

Since we are using a Poisson likelihood (B.3), denoting $\boldsymbol{\lambda} = \mathbf{r}_0 + \mathbf{A}(\mathbf{c}\odot\mathbf{p}) \in \mathbb{R}^{n_{\mathrm{OSN}}}$, then we have:

$$P(\mathbf{s} \mid \mathbf{c}, \mathbf{p}) = \left(\boldsymbol{\lambda}^{\mathbf{s}}\odot e^{-\boldsymbol{\lambda}}\right)\oslash\mathbf{s}! \tag{B.22}$$

where $\boldsymbol{\lambda}^{\mathbf{s}}$, $e^{-\boldsymbol{\lambda}}$ are all element-wise operations. Notably, although the Poisson PMF is discontinuous with respect to $\mathbf{s}$, it is continuous and hence differentiable with respect to $\mathbf{c}$ and $\mathbf{p}$. Therefore, we proceed with:

$$\log P(\mathbf{s} \mid \mathbf{c}, \mathbf{p}) = \log\left(\boldsymbol{\lambda}^{\mathbf{s}}\right) + \log\left(e^{-\boldsymbol{\lambda}}\right) - \log\mathbf{s}! \tag{B.23}$$

$$\boldsymbol{\nabla}_{\mathbf{c}}\log P(\mathbf{s} \mid \mathbf{c}, \mathbf{p}) = (\mathbf{s}\oslash\boldsymbol{\lambda})\odot\boldsymbol{\nabla}_{\mathbf{c}}\boldsymbol{\lambda} - \boldsymbol{\nabla}_{\mathbf{c}}\boldsymbol{\lambda} \tag{B.24}$$

$$\boldsymbol{\nabla}_{\mathbf{c}}\boldsymbol{\lambda} = \boldsymbol{\nabla}_{\mathbf{c}}\left(\mathbf{r}_0 + \mathbf{A}(\mathbf{c}\odot\mathbf{p})\right) = \mathbf{A}^\top(\mathbf{p}\odot(\mathbf{h}-\mathbf{1})) \tag{B.25}$$

Denote $\mathbf{h} = \mathbf{s}\oslash\boldsymbol{\lambda} = \mathbf{s}\oslash(\mathbf{r}_0 + \mathbf{A}(\mathbf{c}\odot\mathbf{p}))$, then we have

$$\boldsymbol{\nabla}_{\mathbf{c}}\log P(\mathbf{s}|\mathbf{p}, \mathbf{c}) = \mathbf{p}\odot[\mathbf{A}^\top(\mathbf{h}-\mathbf{1})] \tag{B.26}$$

Following the same procedure, we have the gradient with respect to $\mathbf{p}$:

$$\boldsymbol{\nabla}_{\mathbf{p}}\log P(\mathbf{s}|\mathbf{p}, \mathbf{c}) = \mathbf{c}\odot[\mathbf{A}^\top(\mathbf{h}-\mathbf{1})] \tag{B.27}$$

bioRxiv preprint doi: https://doi.org/10.1101/2025.11.01.686013; this version posted November 3, 2025. The copyright holder for this preprint (which was not certified by peer review) is the author/funder, who has granted bioRxiv a license to display the preprint in perpetuity. It is made available under aCC-BY 4.0 International license.

Substituting (B.26) and (B.27) back to (B.20), (B.21), we have

$$-\nabla_{\mathbf{c}} V(\mathbf{c},\mathbf{p}) = \mathbf{p} \odot [\mathbf{A}^\top(\mathbf{h}-\mathbf{1})] + \nabla_{\mathbf{c}} \log P(\mathbf{c} \mid \mathbf{p}) \tag{B.28}$$

$$-\nabla_{\mathbf{p}} V(\mathbf{c},\mathbf{p}) = \mathbf{c} \odot [\mathbf{A}^\top(\mathbf{h}-\mathbf{1})] + \nabla_{\mathbf{p}} \log P(\mathbf{c} \mid \mathbf{p}) + \nabla_{\mathbf{p}} \log P(\mathbf{p}) \tag{B.29}$$

We leave the prior terms unspecified as we will subsequently explore different choices of prior.

B.3 Model equations for a continuous Bernoulli prior on presence and a Gamma prior on concentration

We now write down the version of our model that follows the setup in Section 2, *i.e.*, using a continuous Bernoulli prior on presence and a Gamma prior on concentration. As introduced in the main text, we assume that different odorants are independent and identically distributed under the prior. This could be relaxed, but would require more notation. With the continuous relaxation of the presence variables, we choose as our prior a continuous Bernoulli distribution

$$P(p_i) = \frac{1}{Z(\varpi)} \varpi^{p_i}(1-\varpi)^{1-p_i}, \tag{B.30}$$

where $\varpi \in (0,1)$ is a parameter, and $Z(\varpi)$ is a normalization constant. If we were using binary presence variables, we would simplify have $Z(\varpi) = 1$, but in the continuous case we have

$$Z(\varpi) = \int_0^1 \varpi^p (1-\varpi)^{1-p}\, dp = \frac{1-2\varpi}{\log(1/\varpi - 1)}. \tag{B.31}$$

As only the score $\frac{\partial}{\partial p_i} \log P(p_i)$ appears in the dynamics, this normalization constant is for us largely irrelevant.

As introduced in Section 2 of the main text, we choose the prior on $c_i \mid p_i$ to be the same for $p_i = 0$ and $p_i = 1$, as the final estimate $c_i p_i$ is independent of the value of c_i when $p_i = 0$. This choice was also made by Grabska-Barwińska *et al.* [29]. Following those authors, we use a Gamma distribution with parameters α and β:

$$P(c_i | p_i) = \mathrm{Gamma}(c_i\, | \alpha, \beta). \tag{B.32}$$

Now we consider the two components of the prior. Clearly, we have $\nabla_{\mathbf{c}} \log P(\mathbf{p}) = \mathbf{0}$, while

$$\nabla_{\mathbf{p}} \log P(\mathbf{p}) = \log\left(\frac{\varpi}{1-\varpi}\right)\mathbf{1}. \tag{B.33}$$

The log-prior on concentration is

$$\log P(\mathbf{c} \mid \mathbf{p}) = \log\left(\frac{\beta^\alpha}{\Gamma(\alpha)}\right)\mathbf{1} + (\alpha-1)\log \mathbf{c} - \beta \mathbf{c}. \tag{B.34}$$

Because of our simplifying choice that the concentration prior does not depend on presence, we have

$$\nabla_{\mathbf{p}} \log P(\mathbf{c} \mid \mathbf{p}) = 0, \tag{B.35}$$

while the derivative with respect to $\mathbf{c}$ yields

$$\nabla_{\mathbf{c}} \log P(\mathbf{c} \mid \mathbf{p}) = (\alpha-1)\mathbf{1} \oslash \mathbf{c} - \beta \mathbf{1}. \tag{B.36}$$

Substituting (B.33), (B.35) and (B.36) into (B.28) and (B.29), we then have

$$-\nabla_{\mathbf{p}} V = \mathbf{c} \odot [\mathbf{A}^\top(\mathbf{h}-\mathbf{1})] + \log\left(\frac{\varpi}{1-\varpi}\right)\mathbf{1} \tag{B.37}$$

$$-\nabla_{\mathbf{c}} V = \mathbf{p} \odot \mathbf{A}^\top(\mathbf{h}-\mathbf{1}) + (\alpha-1)\mathbf{1} \oslash \mathbf{c} - \beta \mathbf{1} \tag{B.38}$$

Substituting (B.37) and (B.38) into the derived mirrored Langevin dynamics (B.16) and (B.17) respectively, we obtain

$$d\mathbf{u}(t) = \gamma \left[\mathbf{p} \odot (\mathbf{1}-\mathbf{p}) \odot \left(\mathbf{c} \odot [\mathbf{A}^\top(\mathbf{h}-\mathbf{1})] + \log\left(\frac{\varpi}{1-\varpi}\right)\mathbf{1}\right) - 2\mathbf{p} + \mathbf{1}\right] dt + \sqrt{2} d\mathbf{B}_u(t) \tag{B.39}$$

$$d\mathbf{c}(t) = \left[\mathbf{p} \odot [\mathbf{A}^\top(\mathbf{h}-\mathbf{1}) + (\alpha-1)\mathbf{1} \oslash \mathbf{c} - \beta\mathbf{1}]\right] dt + \sqrt{2} d\mathbf{B}_c(t) \tag{B.40}$$

where $\mathbf{h} = \mathbf{s} \oslash (\mathbf{r}_0 + \mathbf{A}(\mathbf{c} \odot \mathbf{p}))$ and $\mathbf{p} = \sigma_\gamma(\mathbf{u})$. This is the soft-gated version of the model.

As the presence $\mathbf{p}$ is essentially binary, we hard gate the dynamics of $\mathbf{c}$. We set $\tilde{p} = 1$ when $p \geq p_{th}$ and $\tilde{p} = 0$ when $p < p_{th}$. This gating can be encoded by $\tilde{\mathbf{p}} = \arg\max_{k \in \{0,1\}} [k\mathbf{p} + (1-k)\mathbf{p}_{th}]$. Replacing $\mathbf{p}$ with $\tilde{\mathbf{p}}$ in (B.40), while

bioRxiv preprint doi: https://doi.org/10.1101/2025.11.01.686013; this version posted November 3, 2025. The copyright holder for this preprint (which was not certified by peer review) is the author/funder, who has granted bioRxiv a license to display the preprint in perpetuity. It is made available under aCC-BY 4.0 International license.

keeping (B.39) unchanged, we have the dynamics of $\mathbf{c}$ with hard gated presence:

$$d\mathbf{c}(t) = \left[\tilde{\mathbf{p}} \odot [\mathbf{A}^\top(\tilde{\mathbf{h}} - \mathbf{1}) + (\alpha - 1)\mathbf{1} \oslash \mathbf{c} - \beta\mathbf{1}]\right] dt + \sqrt{2}d\mathbf{B}_c(t) \tag{B.41}$$

where $\tilde{\mathbf{p}} = \arg\max_{k\in\{0,1\}} [k\mathbf{p} + (1-k)\mathbf{p}_{th}]$ and $\tilde{\mathbf{h}} = \mathbf{s} \oslash (\mathbf{r}_0 + \mathbf{A}(\mathbf{c} \odot \tilde{\mathbf{p}})$.

The SDEs (B.39) and (B.41) represent the full dynamics of our model when using a continuous Bernoulli prior on presence and a Gamma prior on concentration with hard-gating. This variation of our model is the basic version, while further variations are introduced in the following sections.

B.4 Circuit implementation of the model

To implement the coupled dynamics in a biologically plausible way, we first linearize the dynamics using the method proposed in Chalk et al [55] following our previous work [39]. We first introduce a population $\mathbf{h}$ of n_{OSN} neurons with the following dynamics

$$d\mathbf{h}(t) = \mathbf{s} - \mathbf{h} \odot (\mathbf{r}_0 + \mathbf{A}\,(\mathbf{c} \odot \mathbf{p})) \tag{B.42}$$

with a fixed point that is

$$\mathbf{h}^* = \mathbf{s} \oslash (\mathbf{r}_0 + \mathbf{A}(\mathbf{c} \odot \mathbf{p}) \tag{B.43}$$

and a population $\widetilde{\mathbf{h}}$ of n_{OSN} neurons, whose dynamics are

$$d\widetilde{\mathbf{h}}(t) = \mathbf{s} - \mathbf{h} \odot (\mathbf{r}_0 + \mathbf{A}\,(\mathbf{c} \odot \widetilde{\mathbf{p}})) \tag{B.44}$$

with a fixed point that is

$$\widetilde{\mathbf{h}}^* = \mathbf{s} \oslash (\mathbf{r}_0 + \mathbf{A}(\mathbf{c} \odot \widetilde{\mathbf{p}}) \tag{B.45}$$

Then we introduce another population z of $\mathbf{n}_{\text{odor}}$ neurons, whose dynamics are

$$d\mathbf{z}(t) = \boldsymbol{\alpha} - 1 - \mathbf{z} \odot (\boldsymbol{\Gamma}\mathbf{g}) \tag{B.46}$$

with a fixed point that is

$$\mathbf{z}^* = (\boldsymbol{\alpha} - 1) \oslash \mathbf{c} \tag{B.47}$$

Putting everything together, we have the full dynamics.

$$\mathbf{p}(t) = \sigma_\gamma(\mathbf{u}(t)) \tag{B.48}$$

$$\tau_c d\mathbf{c}(t) = \left[\tilde{\mathbf{p}} \odot [\mathbf{A}^\top(\tilde{\mathbf{h}} - \mathbf{1}) + \mathbf{z} - \beta\mathbf{1}]\right] dt + \sqrt{2}d\mathbf{B}_c(t) \tag{B.49}$$

$$\tau_u d\mathbf{u}(t) = \gamma\left[\mathbf{p} \odot (\mathbf{1} - \mathbf{p}) \odot \left(\mathbf{c} \odot [\mathbf{A}^\top(\mathbf{h} - \mathbf{1})] + \log\left(\frac{\varpi}{1-\varpi}\right)\mathbf{1}\right) - 2\mathbf{p} + \mathbf{1}\right] dt + \sqrt{2}d\mathbf{B}_u(t) \tag{B.50}$$

$$\tau_h d\mathbf{h}(t) = \mathbf{s} - \mathbf{h} \odot (\mathbf{r}_0 + \mathbf{A}\,(\mathbf{c} \odot \mathbf{p})) \tag{B.51}$$

$$\tau_{\tilde{h}} d\tilde{\mathbf{h}}(t) = \mathbf{s} - \tilde{\mathbf{h}} \odot (\mathbf{r}_0 + \mathbf{A}\,(\mathbf{c} \odot \tilde{\mathbf{p}})) \tag{B.52}$$

$$\tau_z d\mathbf{z}(t) = \boldsymbol{\alpha} - 1 - \mathbf{z} \odot \mathbf{c} \tag{B.53}$$

We can map these dynamics on the circuit architecture of the olfactory bulb [7, 56]. As they are excited by the OSN input, we interpret $\mathbf{h}$ and $\tilde{\mathbf{h}}$ as the two classes of projection neurons in teh OB (mitral and tufted cells). Then, the concentration estimate $\mathbf{c}$ and presence estimate $\mathbf{p}$ are encoded by local interneurons (granules cells), which inhibit the projection neurons and gate each other's dynamics. The $\mathbf{z}$ neurons required to linearize the prior can then be interpreted as a form of cortical feedback onto the granule cells.

bioRxiv preprint doi: https://doi.org/10.1101/2025.11.01.686013; this version posted November 3, 2025. The copyright holder for this preprint (which was not certified by peer review) is the author/funder, who has granted bioRxiv a license to display the preprint in perpetuity. It is made available under aCC-BY 4.0 International license.

C Kumaraswamy Prior on Presence

In our olfactory sensing model, a prior distribution that reflects the underlying structure of the natural odorants landscape yields a more informative posterior and is expected to promote more efficient inference. Earlier, we used a unimodal continuous Bernoulli (CB) distribution as the prior on presence. However, the nature of the presence variable is bimodal, where p_i tends to be either 0 or 1. Concretely, the natural density of p_i should not be monotonically decreasing near the upper boundary 1, whereas in a Bernoulli prior the density taper off as $p_i \to 1$ (Figure S7a). Therefore, compared to a unimodal prior, a bimodal prior is more realistic, and this motivates us to adopt a bimodal prior distribution.

A commonly used bimodal distribution is the Beta distribution with a correct choice of parameters. However, practically it is hard to adopt the Beta distribution because its density and cumulative distribution function involve non-elementary functions. Fortunately, there is a Beta-type distribution that is easier to work with: the Kumaraswamy (KS) distribution, originally proposed by Kumaraswamy in 1980 [92].

The original KS distribution. A random variable X has the KS distribution if X has density function

$$f_X(x) = abx^{a-1}(1-x^a)^{b-1} \tag{C.1}$$

and cumulative distribution function (CDF)

$$F_X(x) = 1-(1-x^a)^b \tag{C.2}$$

where $a, b > 0$ are shape parameters. The KS distribution, similar to the Beta distribution, can be bimodal, when $a < 1$ and $b < 1$, as visualized in Figure S7a. On the other hand, the KS distribution is also much easier to work with than the Beta distribution for several reasons. One of them is that it only involves simple functions in its density function [93].

Truncation of the KS distribution. The support of the original KS distribution is $(0, 1)$; our presence variable p_i of each odorant, however, is defined on $[0, 1]$. Hence, we must transform variables to ensure that the support of the prior distribution matches the domain of p_i.

To eliminate the asymptotic behavior of the KS distribution near 0 and 1, we first restrict the support of KS distribution to the close interval $C = [0+\epsilon, 1-\epsilon]$ for a small $\epsilon > 0$.

$$f_X^C(x) = f_X(x|x \in C) = \frac{abx^{a-1}(1-x^a)^{b-1}\mathbf{1}(x \in C)}{\int_C abx^{a-1}(1-x^a)^{b-1}dx} \tag{C.3}$$

$$= \frac{abx^{a-1}(1-x^a)^{b-1}\mathbf{1}(x \in C)}{F_X(1-\epsilon)-F_X(\epsilon)} \quad \text{for } x \in (0,1). \tag{C.4}$$

The restricted KS distribution has compact (closed and bounded) support $[0+\epsilon, 1-\epsilon]$ and Lipschitz smooth density function.

We then remap the restricted KS distribution to the full interval $[0, 1]$. We define a function $g(x) = \frac{x-\epsilon}{1-2\epsilon}$ where $\epsilon \in (0, 0.5)$. Given X is a random variable with density function $f_X^C(x)$, we define a new random variable Y as $Y = g(X)$. Since the function g is injective, differentiable and has positive derivative, we can use the change of variables formula to get the probability density function of Y: $f_Y(y) = f_X^C(g^{-1}(y)) \left|\frac{d}{dy}g^{-1}(y)\right|$. Since

$$g^{-1}(y) = y(1-2\epsilon)+\epsilon, \tag{C.5}$$

we have

$$f_Y(y) = \frac{f_X^C(g^{-1}(y))}{1-2\epsilon} = \frac{(1-2\epsilon)\cdot ab\left[g^{-1}(y)\right]^{a-1}\left(1-\left[g^{-1}(y)\right]^a\right)^{b-1}}{F_X(1-\epsilon)-F_X(\epsilon)} \tag{C.6}$$

where $y \in [0, 1]$. Denoting the normalization factor $\alpha = \frac{1-2\epsilon}{F_X(1-\epsilon)-F_X(\epsilon)}$, we have:

$$f_Y(y) = \alpha \cdot ab\left[g^{-1}(y)\right]^{a-1}\left(1-\left[g^{-1}(y)\right]^a\right)^{b-1} \tag{C.7}$$

$$\log f_Y(y) = \log\alpha + \log(ab) + (a-1)\log\left(g^{-1}(y)\right) + (b-1)\log\left(1-\left[g^{-1}(y)\right]^a\right) \tag{C.8}$$

The density function $f_Y(y)$ defines a new probability distribution that is well-defined on $[0, 1]$. and we call this distribution the transformed-KS (TKS) distribution.

bioRxiv preprint doi: https://doi.org/10.1101/2025.11.01.686013; this version posted November 3, 2025. The copyright holder for this preprint (which was not certified by peer review) is the author/funder, who has granted bioRxiv a license to display the preprint in perpetuity. It is made available under aCC-BY 4.0 International license.

It follows that if we let $z = g^{-1}(y) = y(1-2\epsilon)+\epsilon$, the first and second derivative of the log distribution (C.8) are:

$$\nabla \log f_Y(y) = (1-2\epsilon)\left[\frac{a-1}{z} - \frac{a(b-1)z^{a-1}}{1-z^a}\right] \tag{C.9}$$

$$\nabla^2 \log f_Y(y) = (1-2\epsilon)^2\left[-\frac{a-1}{z^2} - a(b-1)\frac{(a-1)z^{a-2}+z^{2a-2}}{(1-z^a)^2}\right] \tag{C.10}$$

The first and second derivative of the log distribution are visualized in the third and fourth columns (left-to-right) in Figure S7a.

We now choose a particular TKS distribution with the set of parameters a, b and ϵ to be the prior on presence. As before, we denote the prior distribution density function as $P(\mathbf{p})$ in vector form. The gradient of the log-prior with respect to $\mathbf{c}$ is 0, while the derivative with respect to $\mathbf{p}$ is

$$\boldsymbol{\nabla}_{\mathbf{p}} \log P(\mathbf{p}) = (1-2\epsilon)\left[(ab-1)\left(g^{-1}(\mathbf{p})\right)^a - a + 1\right] \oslash \left[g^{-1}(\mathbf{p}) \odot \left(\left(g^{-1}(\mathbf{p})\right)^a - 1\right)\right] \tag{C.11}$$

where $g(\mathbf{p}) = (\mathbf{p}-\epsilon) \oslash (1-2\epsilon)$ is the remapping function. The above equation (C.11) directly follows from (C.8).

Using (C.9), the gradient of the energy function with respect to $\mathbf{p}$ is

$$-\boldsymbol{\nabla}_{\mathbf{p}} V = \mathbf{c} \odot \left[\mathbf{A}^\top(\mathbf{h}-\mathbf{1})\right] + (1-2\epsilon)\left[(ab-1)\left(g^{-1}(\mathbf{p})\right)^a - a + 1\right] \oslash \left[g^{-1}(\mathbf{p}) \odot \left(\left(g^{-1}(\mathbf{p})\right)^a - 1\right)\right] \tag{C.12}$$

while the gradient with respect to $\mathbf{c}$ is unchanged:

$$-\boldsymbol{\nabla}_{\mathbf{c}} V = \mathbf{p} \odot \mathbf{A}^\top(\mathbf{h}-\mathbf{1}) + (\alpha-1)\mathbf{1} \oslash \mathbf{c} - \beta\mathbf{1} \tag{C.13}$$

Thus, the full dynamics yields

$$d\mathbf{u}(t) = \gamma\Big[\mathbf{p} \odot (\mathbf{1}-\mathbf{p}) \odot \Big(\mathbf{c} \odot \left[\mathbf{A}^\top(\mathbf{h}-\mathbf{1})\right] + (1-2\epsilon)\frac{(ab-1)\left(g^{-1}(\mathbf{p})\right)^a - a + 1}{g^{-1}(\mathbf{p}) \odot \left((g^{-1}(\mathbf{p}))^a - 1\right)}\Big) - 2\mathbf{p} + \mathbf{1}\Big]\,dt \tag{C.14}$$

$$+ \sqrt{2}\,d\mathbf{B}_u(t). \tag{C.15}$$

and

$$d\mathbf{c}(t) = \Big[\mathbf{p} \odot \left[\mathbf{A}^\top(\mathbf{h}-\mathbf{1}) + (\alpha-1)\mathbf{1} \oslash \mathbf{c} - \beta\mathbf{1}\right]\Big]\,dt + \sqrt{2}d\mathbf{B}_c(t). \tag{C.16}$$

We now try to gain some more intuition for the behavior of the prior over $\mathbf{u}$ resulting from the truncated KS prior over $\mathbf{p}$. Importantly, it is clear that it is not log-concave. As the prior is factorized over odorants, consider the j-th odorant, for which we have:

$$W(u_j) \equiv \log P(p_j(u_j)) = C + (a-1)\log\left[\frac{1-2\epsilon}{1+e^{-\gamma u_j}} + \epsilon\right] + (b-1)\log\left(1 - \left[\frac{1-2\epsilon}{1+e^{-\gamma u_j}} + \epsilon\right]^a\right) \tag{C.17}$$

where C is a normalizing constant. We can also derive $W'(u_j)$ and $W''(u_j)$ as following:

$$W'(u_j) = \frac{d}{du}\log P(p_j(u_j)) = \frac{d}{du}p_j(u_j) \cdot \frac{d}{dp_j}\log P(p_j(u_j)) \tag{C.18}$$

$$= \gamma \cdot p_j(u_j)(1-p_j(u_j)) \cdot (1-2\epsilon)\left[\frac{a-1}{p_j(u_j)} - \frac{a(b-1)p_j(u_j)^{a-1}}{1-p_j(u_j)^a}\right], \tag{C.19}$$

where we get $\frac{d}{dp_j}\log P(p_j(u_j))$ from (C.9).

$$W''(u_j) = \frac{d^2}{du^2}\log P(p_j(u_j)) = \frac{d}{du}\left[\frac{d}{du}p_j(u_j) \cdot \frac{d}{dp_j}\log P(p_j(u_j))\right] \tag{C.20}$$

$$= \frac{d^2}{dp_j^2}\log P(p_j(u_j)) \cdot \left(\frac{d}{du}p_j(u_j)\right)^2 + \frac{d}{dp_j}\log P(p_j(u_j)) \cdot \frac{d^2}{du^2}p_j(u_j) \tag{C.21}$$

We can then substitute (C.9) and (C.10) in and get the second derivative w.r.t. u_j.

For any positive small $1 \gg \epsilon > 0$, $W(u_j)$ is roughly constant for any u_j of even modestly large absolute value, with a transition region between. In particular, for $u_j \to -\infty$ we have

$$W(u_j) \approx C + (a-1)\log(\epsilon) + (b-1)\log(1-\epsilon^a) \tag{C.22}$$

bioRxiv preprint doi: https://doi.org/10.1101/2025.11.01.686013; this version posted November 3, 2025. The copyright holder for this preprint (which was not certified by peer review) is the author/funder, who has granted bioRxiv a license to display the preprint in perpetuity. It is made available under aCC-BY 4.0 International license.

while for $u_j \to \infty$ we have

$$W(u_j) \approx C + (a-1)\log(1-\epsilon) + (b-1)\log[1-(1-\epsilon)^a]. \tag{C.23}$$

In between, we note that

$$W(u_j) = C + (1-a)\log(2) + (b-1)\log(1-2^{-a}). \tag{C.24}$$

What remains is to figure out the behavior of the function in the transition region, as well as the width of that region. By direct computation, we find that the only stationary point u_j^* of $W(u_j)$ (that is, the solution to $W'(u_j^*) = 0$) is

$$u_j^* = \frac{1}{\gamma}\log\frac{\beta-\epsilon}{1-\beta-\epsilon} \quad \text{where} \quad \beta = \left(\frac{1-a}{1-ab}\right)^{1/a}. \tag{C.25}$$

Using Mathematica, we can verify that the Hessian $W''(u_j^*) > 0$ at this point, meaning that it is a local minimum. Moreover, we can see that $W'(u_j) > 0$ for $u_j > u_j^*$, while $W'(u_j) < 0$ for $u_j < u_j^*$. Thus, the two plateaus are separated by a low-probability well. By inspection, and from Figure S7c, we can see also that the Hessian $W''(u_j)$ does not have definite sign, meaning that the prior is not log-concave.

In addition, the necessity to truncate the original distribution becomes obvious as we compare between Figure S7c&d. The log prior distribution as a function of u_j is unbounded on $\mathbb{R}$, causing numerical instability in implementation.

Re-parametrization of the original KS distribution. To select the desired parameters a, b, we introduce a reparameterization of the original KS distribution PDF . Specifically, we re-parameterize the PDF w.r.t a, r instead of the original a, b. We start from the CDF of the original KS distribution (C.2) and set $F_X(0.5) = P(X \leq 0.5) = r$:

$$1-(1-0.5^a)^b = r \quad \Longrightarrow \quad b = \frac{\log(1-r)}{\log 1 - 0.5^a} \tag{C.26}$$

Hence we have $KS_{reparam}(a,r) = KS(a, \frac{\log(1-r)}{\log 1-0.5^a})$. Under this parameterization, we interpret a as a shape parameter and r as a bias parameter the balance between the density near the two end points. This parameterization is much easier to interpret and useful when finding a desired distribution.

Although we lose the exact relation that $P(X \leq 0.5) = r$ after the truncation process, the qualitative properties of the distribution is mostly unchanged. Hence, this reparameterization is still helpful for selecting parameter for truncated KS distribution. Particularly, in the simulations, we let $a = 0.055$ and $r = 0.75$, which yields $(a, b) = (0.055, 0.422)$ in the original expression.

D Presence-dependent concentration priors

For most of this paper, we have imposed the simplifying assumption that the conditional prior on concentration is the same irrespective of whether or not an odorant is present. However, such a formalism yields an artifact that our latent concentration estimates c_i does not goes to 0 when $p_i = 0$. To address this, we introduce a presence-dependent concentration prior that favors having $c_i = 0$ when $p_i = 0$. We design a differentiable prior composed of a sparsity-encouraging exponential distribution (that is, an L_1 penalty) with rate λ when $p_i = 0$, and a Gamma distribution with parameters α and β otherwise. This is defined as:

$$P(c_i|p_i) = (1-p_i)\lambda e^{-\lambda c_i} + p_i \,\mathrm{Gamma}(c_i\,|\alpha,\beta). \tag{D.1}$$

It follows that the log prior is

$$\log P(\mathbf{c} \mid \mathbf{p}) = (1-\mathbf{p}) \odot [\log(\lambda)\mathbf{1} - \lambda\mathbf{c}] + \mathbf{p} \odot \left[\log\left(\frac{\beta^\alpha}{\Gamma(\alpha)}\right)\mathbf{1} + (\alpha-1)\log\mathbf{c} - \beta\mathbf{c}\right]. \tag{D.2}$$

Its derivatives with respect to $\mathbf{p}$ and $\mathbf{c}$ are

$$\nabla_{\mathbf{p}} \log P(\mathbf{c} \mid \mathbf{p}) = -[\log(\lambda)\mathbf{1} - \lambda\mathbf{c}] + \left[\log\left(\frac{\beta^\alpha}{\Gamma(\alpha)}\right)\mathbf{1} + (\alpha-1)\log\mathbf{c} - \beta\mathbf{c}\right] \tag{D.3}$$

$$= -\log\left(\frac{\lambda\Gamma(\alpha)}{\beta^\alpha}\right)\mathbf{1} + (\alpha-1)\log\mathbf{c} - (\beta-\lambda)\mathbf{c}, \tag{D.4}$$

bioRxiv preprint doi: https://doi.org/10.1101/2025.11.01.686013; this version posted November 3, 2025. The copyright holder for this preprint (which was not certified by peer review) is the author/funder, who has granted bioRxiv a license to display the preprint in perpetuity. It is made available under aCC-BY 4.0 International license.

and

$$\nabla_{\mathbf{c}} \log P(\mathbf{c} \mid \mathbf{p}) = -\lambda(\mathbf{1} - \mathbf{p}) + \mathbf{p} \odot [(\alpha - 1)\mathbf{1} \oslash \mathbf{c} - \beta \mathbf{1}], \tag{D.5}$$

respectively. With the same definition of the energy function as before, we have

$$-\nabla_{\mathbf{p}} V = \mathbf{c} \odot [\mathbf{A}^\top(\mathbf{h} - \mathbf{1})] - \rho \mathbf{1} + (\alpha - 1)\log \mathbf{c} + (\lambda - \beta)\mathbf{c} \tag{D.6}$$

and

$$-\nabla_{\mathbf{c}} V = \mathbf{p} \odot [\mathbf{A}^\top(\mathbf{h} - \mathbf{1}) + (\alpha - 1)\mathbf{1} \oslash \mathbf{c} - \beta \mathbf{1}] - \lambda(\mathbf{1} - \mathbf{p}), \tag{D.7}$$

where we now let

$$\rho = -\log\left(\frac{\varpi}{1-\varpi}\frac{\beta^\alpha}{\lambda\Gamma(\alpha)}\right). \tag{D.8}$$

Thus, the full SDEO model dynamics with this prior are

$$d\mathbf{u}(t) = \gamma\left[\mathbf{p} \odot (\mathbf{1} - \mathbf{p}) \odot \Big(\mathbf{c} \odot [\mathbf{A}^\top(\mathbf{h} - \mathbf{1})] - \rho\mathbf{1} + (\alpha - 1)\log \mathbf{c} + (\lambda - \beta)\mathbf{c}\Big) - 2\mathbf{p} + \mathbf{1}\right] dt + \sqrt{2}d\mathbf{B}_u(t) \tag{D.9}$$

and

$$d\mathbf{c}(t) = \Big[\mathbf{p} \odot [\mathbf{A}^\top(\mathbf{h} - \mathbf{1}) + (\alpha - 1)\mathbf{1} \oslash \mathbf{c} - \beta\mathbf{1}] - \lambda(\mathbf{1} - \mathbf{p})\Big]\, dt + \sqrt{2}d\mathbf{B}_c(t). \tag{D.10}$$

We show an example simulation of these dynamics in Figure S3. In contrast to Figures 3, S1, and S2, we see that with this choice of prior the latent concentration estimates for non-present odors decay to zero once the presence estimate indicates that the odor is absent. Moreover, in the dynamics above, we see that for $\alpha > 1$ the update to the presence estimate coming from the conditional prior on $\mathbf{c} \mid \mathbf{p}$ has an interesting effect: it will tend to push low-$\mathbf{c}$ odors towards being recognized as absent, as $(\alpha - 1)\log \mathbf{c}$ will become strongly negative.

E Mutual coherence of affinity matrix and scaling capacity

In this section we will discuss our model from a pure compressive sensing perspective, focusing on how the properties of the random sensing matrix—in our case, the affinity matrix—affect the scaling capacity. The key property of interest is the *mutual coherence* of the sensing matrix, which is one measure of how closely the measurement approximates an isometry. It is important to note that compressed sensing with Poisson noise is not as well-understood as the standard case of additive Gaussian noise [27, 94]. However, many of the same desiderata for the sensing matrix carry over. Namely, the mutual coherence should ideally be small.

The mutual coherence is a measure of the worst-case similarity between the columns of a projection matrix $\boldsymbol{A}$. It is defined as:

$$\mu\{\boldsymbol{A}\} = \max_{i \neq j} \frac{|\langle \boldsymbol{a}_i, \boldsymbol{a}_j \rangle|}{\|\boldsymbol{a}_i\|_2 \|\boldsymbol{a}_j\|_2}, \tag{E.1}$$

where $\boldsymbol{a}_i$ is the i-th column of $\mathbf{A}$. Equivalently, the mutual coherence is the maximum absolute value of the off-diagonal elements of the normalized Gram matrix

$$G_{ij} = \frac{\langle \boldsymbol{a}_i, \boldsymbol{a}_j \rangle}{\|\boldsymbol{a}_i\|_2 \|\boldsymbol{a}_j\|_2}, \tag{E.2}$$

i.e., $\mu\{\boldsymbol{A}\} = \max_{i \neq j} |G_{ij}|$. To achieve good compressed sensing performance, the mutual coherence should be small.

However, the mutual coherence is a worst-case measure, and thus can give extremely pessimistic predictions relative to the performance of a particular sensing matrix in practice. On these grounds, Elad [61] argued that the *t-averaged mutual coherence*

$$\mu_t\{A\} = \frac{\sum_{i \neq j} \mathbf{1}(|G_{ij}| \geq t)|G_{ij}|}{\sum_{i \neq j} \mathbf{1}(|G_{ij}| \geq t)} \tag{E.3}$$

can provide a more informative measure. Here, $\mathbf{1}(\cdot)$ is an indicator function which is equal to one when the predicate in its argument is true, and zero otherwise.

Using these two measures, we can evaluate the quality of an affinity matrix $\boldsymbol{A}$. Particularly, we will use the *t-averaged*

bioRxiv preprint doi: https://doi.org/10.1101/2025.11.01.686013; this version posted November 3, 2025. The copyright holder for this preprint (which was not certified by peer review) is the author/funder, who has granted bioRxiv a license to display the preprint in perpetuity. It is made available under aCC-BY 4.0 International license.

mutual coherence, where the t value is set to be the 80% quantile of the Gram matrix, which gives us the average of top 20% worst cases.

We are interested in three ensembles of random affinity matrices:

1. **Dense gamma affinity matrix**: each element of the affinity matrix follows a Gamma distribution, and the matrix is normalized by its largest element;

2. **Sparse binary affinity matrix**: each element of the affinity matrix follows a Bernoulli distribution, being 1 with a probability p and 0 otherwise;

3. **Sparse gamma affinity matrix**: each element of the affinity matrix follows Gamma distribution with probability p and 0 otherwise.

We plot examples of these three types of sensing matrices in Figure S8a.

In Figures 7 and S6, we saw that sparse binary affinity matrices seem in general to achieve the highest capacity, followed by sparse gamma and then dense gamma. Examining the mutual coherence distributions for each of these ensembles, we see that a moderate system size (dictionary of 5000 odorants, 600 sensors), we notice that the sparse Gamma sensing matrix has the lowest average mutual coherence μ_t but the sparse binary sensing matrix has the lowest mutual coherence μ (Figure S8b). The average mutual coherence varies with the dictionary size and with the number of sensors, but this ordering remains roughly consistent across scales (Figure S8c).

In the sparse sensing ensembles, a crucial parameter governing the mutual coherence is the mean sparsity (average fraction of non-zero elements) p [27]; sparser matrices have lower average mutual coherence (Figure S8d). As we would expect, this results in a scaling capacity that decreases with increasing p across a variety of dictionary and sensor repertoire sizes (Figure S8e-f). Therefore, our empirical results are consistent with the broad conclusion that lower average mutual coherence enables higher compressive sensing capacity.

F Numerical methods and additional scaling results

F.1 General numerical method pipeline

This section illustrates the general numerical analysis pipeline used in this paper. As an example, we showed how we numerically simulate the SDEs (B.39), (B.41) and (C.14). These equations describe the full model dynamics under the assumption of 1-on-1 coding in granule cells. They use either the Bernoulli prior (B.39) or the Kumaraswamy prior (C.14) on the presence.

We first discretized the time domain by considering a step size of $\Delta t = 10^{-5}$, resulting in a total of $N = \frac{T}{\Delta t}$ timesteps. Afterward, applying the Euler-Maruyama method, along with additional treatments to stabilize the numerical computation, we obtained a discrete-time Markov chain $(X_n : 0 \leq n \leq N)$ representing the evolution of the estimated $\mathbf{c}$ and $\mathbf{u}$ over time. Since the subscript n is already used to denote the time step, we use $\mathbf{p}_n[i]$ to denote the i-th element of the vector $\mathbf{p}_n$ in this section.

In particular, we have $X_n = (\mathbf{c}_n, \mathbf{u}_n) \in \mathcal{S} := \mathbb{R}^{n_{\text{odor}}} \times \mathbb{R}^{n_{\text{odor}}}$ with its transition rules given by:

$$\mathbf{c}_{n+1} = \max\{\mathbf{c}'_{n+1}, \varepsilon\} \qquad (n = 0, 1, \ldots, N). \tag{F.1}$$

$$\mathbf{c}'_{n+1} = \mathbf{c}_n + \left[\widetilde{\mathbf{p}}_n \odot \left(\mathbf{A}^\top(\widetilde{\mathbf{h}}_n - \mathbf{1}) + (\alpha - 1)\,\mathbf{1} \oslash (\mathbf{c}_n + \varepsilon) - \beta\,\mathbf{1}\right)\right] \oslash \tau_{g_t} + \sqrt{2}\,\Delta\mathbf{B}_{c,n} \tag{F.2}$$

$$\mathbf{u}_{n+1} = \mathbf{u}_n + \gamma \odot \left[\mathbf{p}_n \odot (\mathbf{1} - \mathbf{p}_n) \odot \left(\mathbf{c}_n \odot \left[\mathbf{A}^\top(\mathbf{h}_n - \mathbf{1})\right] + \nabla_{\mathbf{p}} \log P(\mathbf{p}_n)\right) - 2\,\mathbf{p}_n + \mathbf{1}\right] \oslash \tau_{g_m} + \sqrt{2}\,\Delta\mathbf{B}_{u,n}, \tag{F.3}$$

bioRxiv preprint doi: https://doi.org/10.1101/2025.11.01.686013; this version posted November 3, 2025. The copyright holder for this preprint (which was not certified by peer review) is the author/funder, who has granted bioRxiv a license to display the preprint in perpetuity. It is made available under aCC-BY 4.0 International license.

where

$$\mathbf{p}_n = \sigma_\gamma(\mathbf{u}_n), \tag{F.4}$$

$$\mathbf{h}_n = \mathbf{s} \oslash \Big(\mathbf{r}_0 + \mathbf{A}\,[\,\mathbf{c}_n \odot \mathbf{p}_n\,]\Big), \tag{F.5}$$

$$\widetilde{\mathbf{h}}_n = \mathbf{s} \oslash \Big(\mathbf{r}_0 + \mathbf{A}\,[\,\mathbf{c}_n \odot \widetilde{\mathbf{p}}_n\,]\Big), \tag{F.6}$$

$$\mathbf{s} \sim \text{Poisson}(\mathbf{r}_0 + \mathbf{A}(\mathbf{c}_{\text{True}} \odot \mathbf{p}_{\text{True}})) \tag{F.7}$$

$$\widetilde{\mathbf{p}}_n[i] = \Big(\mathbf{1}_{\{\mathbf{p}_n[i] \geq p_{\text{th}}\}} + \varepsilon\,\mathbf{1}_{\{\mathbf{p}_n[i] < p_{\text{th}}\}}\Big)\,\mathbf{p}_n[i], \quad i = 1, \dots, n_{\text{odor}}, \tag{F.8}$$

$$\Delta B_{c,n} = B_{c,n+1} - B_{c,n} \sim \sqrt{\frac{1}{\tau_{g_t}}} \cdot \mathcal{N}(0, \Delta t), \quad \Delta B_{u,n} = B_{u,n+1} - B_{u,n} \sim \sqrt{\frac{1}{\tau_{g_m}}} \cdot \mathcal{N}(0, \Delta t). \tag{F.9}$$

and

$$\nabla_{\mathbf{p}} \log P(\mathbf{p}_n) = \begin{cases} \log\Big(\dfrac{\varpi}{1-\varpi}\Big)\,\mathbf{1}, & \text{(Bernoulli prior)}, \\ \big[\,(ab-1)\,\mathbf{p}_n^a - (a-1)\,\mathbf{1}\big] \oslash \big[\,\mathbf{p}_n \odot (\mathbf{p}_n^a - \mathbf{1})\big], & \text{(Kumaraswamy prior)}. \end{cases} \tag{F.10}$$

In particular, we used the following hyperparameter settings: $\mathbf{r}_0 = \mathbf{1} \in \mathbb{R}^{n_{\text{OSN}}}$, $\tau_{g_t} = \tau_{g_m} = 0.02$, $\alpha = 5.0$, $\beta = 0.1$, $\gamma = 5.0$ and $\varepsilon = 10^{-5}$. For the Bernoulli prior, we set $\omega = 0.1$ and $p_{th} = 0.2$; for the Kumaraswamy prior, we set $a = 0.1$, $b = 0.5$ and $p_{th} = 0.5$. The affinity matrix $\mathbf{A}$ is one of the three types of random matrix discussed in Appendix E.

Since the Markov chain produced by the Euler-Maruyama method approximates both the trajectory and stationary distribution of the SDEs, its behavior—particularly empirical convergence and convergence rate—reflects the model's capacity to infer the presence and concentration of odorants. In general, analyzing the behavior of this Markov chain suffices as a numerical approach for evaluating the model's system of SDEs.

Unless otherwise specified, the same numerical procedure was applied to variants of the basic model. Modifications specific to each variant are discussed in the corresponding sections.

We summarize all parameter used in the scaling simulation in Table 2.

F.2 Further computational implementation and computing resources

All scaling simulations were implemented in Python 3.11 using the PyTorch framework (version 2.6.0+computecanada) and executed on AMD EPYC 9655 CPU nodes of the Digital Research Alliance of Canada Fir cluster. Several runtime optimizations were applied to improve performance and ensure stability across different CPU types and under varying levels of cluster load.

Because iterated Euler integration involves repeated multiplication of large matrices—an increasingly expensive operation as dimensionality grows—single-simulation runtime optimization focused on efficient matrix computation. We used PyTorch tensors instead of NumPy arrays for most matrix operations to leverage the multi-processing in PyTorch. Further speed-up was obtained by using PyTorch's Just-In-Time (JIT) compilation for the core Euler-forward SDE integration kernels. In addition, we parallelize matrix computations across 4 CPU cores for the baseline model with OpenMP. All of these optimizations yield a 2-3x speedup over vanilla NumPy implementations in high-dimensional settings.

At the experiment level, each sweep experiment was partitioned into multiple jobs and dispatched as independent tasks using SLURM's array feature. File I/O was handled via the h5py library for efficient data storage and convenient retrieval. Additional algorithmic optimizations for scaling capacity experiments are discussed in Appendix F.4.4.

Specific runtime varies across platform. In our case, one simulation with 5×10^4 iteration steps at the highest dimensionality (16K possible odorants) took at most 1200s for SDEO model, and up to ∼7000s for the baseline model. Thus, a significant proportion of the CPU hours was spent running the baseline comparison in Figures 7 and S6. Total compute time required to generate all the figures is ∼12000 CPU hours.

bioRxiv preprint doi: https://doi.org/10.1101/2025.11.01.686013; this version posted November 3, 2025. The copyright holder for this preprint (which was not certified by peer review) is the author/funder, who has granted bioRxiv a license to display the preprint in perpetuity. It is made available under aCC-BY 4.0 International license.

Table 2: Hyperparameter used in simulations

Variable name	Description	Value	Unit
**Single Neuron parameters**			
$\mathbf{r}_0$	Baseline firing rate of OSN	1.0	
τ_c	Time constant for concentration estimation	0.02	second
τ_p	Time constant for presence estimation	0.02	second
**Prior distribution for concentration**			
α	Parameter for $\mathrm{Gamma}(\alpha, \beta)$	5.0	
β	Parameter for $\mathrm{Gamma}(\alpha, \beta)$	0.1	
**Prior distribution for presence**			
ϖ	Parameter for the continuous Bernoulli prior distribution	0.01	
a	Parameter for truncated Kumaraswamy distribution	0.055	
b	Parameter for truncated Kumaraswamy distribution	0.422	
ϵ	Truncation cutoff for truncated Kumaraswamy distribution	1×10^{-5}	
**Presence and u in dual space**			
p_{th}	Threshold used for binarizing the continuously relaxed presence	0.2	
γ	Parameter in the sigmoid function that transform **p** into **u**	5.0	
**Non-separated model**			
τ_E	Time constant for excitatory neurons	0.02	second
τ_I	Time constant for inhibitory neurons	0.03	second
λ	Parameter for the L1 exponential prior distribution	0.03	
**Simulation parameter**			
Δt		1×10^{-5}	second

F.3 Baseline model and metrics for evaluation

F.3.1 Baseline model

To demonstrate the improved capacity of our model through comparison, we use a model we proposed in [39] as a baseline. Specifically, we use the proposed model with one-to-one coding between neurons and odorants. We confirm that the performance of the baseline model we obtain here is consistent with the results in previous work. For validation of consistency, Figures S5 and S6 in this work can be indirectly compared with Figures 3 in [39]. We note that the displayed performance appear worse because we adopt stricter evaluation criteria and shorten available time for inference to ensure comparability with the high performance of the SDEO model. For example, we define correct estimate as one in which the estimated concentration falls within $\pm 25\% \times \text{True Concentration}$, while the previous work use $\pm 50\%$.

F.3.2 Metrics for concentration estimation

We introduce the two metrics for evaluating the accuracy of the concentration inference. Recall that we denote the number of presented odors as n_{present}, for any concentration estimates $\hat{\mathbf{c}}$ given the model, we have:

1. **Mean absolute error:** We compute the mean absolute error (MAE) between inferred and true concentrations for the presented odors: $\mathrm{MAE}_{\hat{\mathbf{c}}} = \frac{1}{n_{\text{present}}} \sum_{i=1}^{n_{\text{present}}} |\hat{\mathbf{c}}[i] - \mathbf{c}_{\text{True}}[i]|$. In Figures 6 and S5, MAE is plotted as curves in panel **a** and as heatmaps in panel **b**.

2. **Correct proportion:** We calculate the proportion of presented odors whose predicted concentrations fall within a $\pm\delta$ neighborhood of the true value: $\rho = \frac{1}{n_{\text{present}}} \sum_{i=1}^{n_{\text{present}}} \mathbf{1}\left(|\hat{\mathbf{c}}[i] - \mathbf{c}_{\text{True}}[i]| < \delta\right)$. The correct proportion computed using $\delta = 10$ is shown as contours in panel **b** of Figures 6 and S5. We note that since we set the true con-

bioRxiv preprint doi: https://doi.org/10.1101/2025.11.01.686013; this version posted November 3, 2025. The copyright holder for this preprint (which was not certified by peer review) is the author/funder, who has granted bioRxiv a license to display the preprint in perpetuity. It is made available under aCC-BY 4.0 International license.

centration value to be 40, the correct proportion with $\delta = 10$ corresponds of the proportion of correct concentration estimates within a tolerance of $\pm 25\% \times$True concentration.

F.3.3 Metrics for presence estimation

To introduce metrics for the quality of the presence inference, we begin by noting that the detection of odorants is inherently a binary classification problem. We therefore adopt a metric, AUROC score, from receiver operating characteristic (ROC) analysis to quantify the probability that the presence of odorants can be decoded from based on the output of the model. In ROC analysis, the ROC curve is the function of the true positive rate (TPR) over the false positive rate (FPR), which can be plotting by interpolating the TPR versus FPR at all possible thresholds. The AUROC score ($AUC \in [0, 1]$) is defined as the area under the ROC curve. It can be computed given estimated value vector $\hat{\mathbf{p}}$ and a ground truth label vector $\mathbf{p}_{\text{True}}$. Importantly, its value is exactly the probability of responding correctly in the two-alternative forced-choice test based on a given data [95], and it reflects the discrimination between the representation of present and absent odorants in $\hat{\mathbf{p}}$.

AUROC score can also be interpreted as the integration of the accuracy over all possible classifying thresholds. We briefly showed that this interpretation aligns with the standard definition of AUROC score. Let z denote the threshold that downstream neurons use to classify $\mathbf{p}$. Define:

- the true positive rate as $\beta(z) = P(p \geq z|\text{present})$
- the probability density function of presence p given the odorant is absent as $f_{abs}(z) = \frac{d}{dz}F_{abs}(z)$ where $F_{abs}(z) = P(p \leq z|\text{absent})$.

We then have the probability of decoding correctly as:

$$P[correct] = \int_{-\infty}^{\infty} \beta(z) f_{abs}(z) dz = \int_{0}^{1} \beta(z) f_{abs}(z) dz \tag{F.11}$$

Now consider the false positive rate $\alpha(z) = P(p \geq z|\text{absent})$, notice that:

$$\alpha(z) = P(p \geq z|\text{absent}) = \int_{z}^{\infty} f_{abs}(r) dr = \int_{z}^{1} f_{abs}(r) dr \Rightarrow \frac{d\alpha}{dz} = -f_{abs}(z) \tag{F.12}$$

Substitute (F.12) into (F.11) we have:

$$P[correct] = \int_{0}^{1} \beta(z) f_{abs}(z) dz = \int_{0}^{1} \beta(z) d\alpha(z) \tag{F.13}$$

Given that the ROC curve is $\beta = \psi(\alpha)$, where $\psi : [0, 1] \mapsto [0, 1]$ maps the FPR α to the TPR β, the right hand side of (F.13) corresponds to the area under the ROC curve. Therefore, the AUROC score is indeed the probability of correct classification in a 2FAC task.

As a direct consequence of its definition, AUROC score is threshold-independent. It also inherently incorporates both TPR and FPR in a single scaler value and reflects the discrimination between the two classes in the data we are performing the classification on. In comparison, most other metrics cannot account for TPR and FPR simultaneously, requiring additional metric as complementary indicator. Therefore, AUROC score provides a more comprehensive assessment of a classifier's performance by considering the probability of correct classification than traditional threshold-based binary classification metrics.

A AUROC score of 0.85 indicates that, for any randomly chosen pair consisting of a present odor and an absent odor, the model will correctly classify the present odor with a probability of 85%. Effectively, this means that, on average, 85% of all odors can be correctly classified, and in particular, 85% of the presented odors can be correctly classified as present. We can see that although the AUROC score captures more information about the model's presence estimation performance, the two metrics have some extent of equivalency in their interpretation, and this allows a clear and direct comparison between the two models.

Hence we use the AUROC score as the primary metric for evaluating the presence estimation performance of our model. In Figures 6 and S5, we plotted the AUROC score as curves in panel **c** and as heatmaps with contours overlaid in panel **d**.

bioRxiv preprint doi: https://doi.org/10.1101/2025.11.01.686013; this version posted November 3, 2025. The copyright holder for this preprint (which was not certified by peer review) is the author/funder, who has granted bioRxiv a license to display the preprint in perpetuity. It is made available under aCC-BY 4.0 International license.

F.3.4 Maximum detection capacity

Lastly, to investigate the scaling properties of the SDEO model, we introduce the *maximum detection capacity* κ to quantify the largest number of simultaneously presented odorants that the model can reliably detect given a certain number of sensors and dictionary size. For a fixed n_{OSN} and n_{odor}, we first define *maximum detection capacity* assessed by presence estimates: κ_{AUROC}. Specifically, we let it to be the largest n_{present} such that the AUROC score on model's presence estimates $\hat{\mathbf{p}}$ remains above a threshold ϵ_t:

$$\kappa_{\text{AUROC}} = \arg\max_{j \in S} \{n_{\text{present}} = j \mid \text{AUROC}(\hat{\mathbf{p}}) \geq \epsilon_t\} \tag{F.14}$$

where $S \subset \mathbb{N}$ denotes the search space. In practice, the search space is chosen to balance computational cost and the resolution. Similarly, we define its counterpart that evaluates the concentration estimates as κ_{MAE}. Notably, since larger MAE implies worse performance, κ_{MAE} is defined by MAE remaining less than some threshold ϵ_t:

$$\kappa_{\text{MAE}} = \arg\max_{j \in S} \{n_{\text{present}} = j \mid \text{MAE}(\hat{\mathbf{c}}) \geq \epsilon_t\} \tag{F.15}$$

In Figures 7 and S8 (panel **f**), we plot the maximum detection capacity evaluated by the AUROC score as heatmaps with contours overlaid. The threshold used is the AUROC score = 0.85.

In Figure S6, we plot the maximum detection capacity evaluated by the MAE as heatmaps with contours overlaid. The threshold used is 10 concentration unit. This represents a 25% relative error tolerance as the true concentration is 40.

F.4 Experimental setting and supplemental figures

F.4.1 Single simulation

Here we explain how a single simulation—the building block of all scaling experiment—is executed.

The first step of each simulation is to generate sensory scene, which includes generating ground truth time series, affinity matrix for OSNs, and multiplying it with ground truth time series to get OSNs responses. We considered the OSN responses $\mathbf{s}$ to be static throughout the inference, hence the values of $\mathbf{s}$ are drawn once from the Poisson distribution in (F.7). Since the affinity matrix is randomly generated in each trial, without loss of generality, we always pick the first n_{present} in the dictionary to be the presented odors in the implementation level.

This is followed by the initialization of network. Each components of the concentration vector $\mathbf{c}_0$ is independently drawn from a Gamma distribution: $\mathbf{c}_0[i] \sim \text{Gamma}(6, 4)$ for $i = 1, \ldots, n_{\text{odor}}$, while each components of the presence vector $\mathbf{p}_0$ was set such that $\frac{1}{1+\exp(-\gamma \cdot \mathbf{u}_0[i])} = \varpi$. Consequently, the initial concentration states are randomly assigned, while the initial presence values correspond to their prior probability ϖ. In our simulations, the prior probability ϖ was set to 0.1 for all odorants. However, these prior need not be identical—they can be assigned heterogeneously or updated adaptively based through a learning process. In fact, non-uniform and adaptive prior may better reflect the implementation in biological system. Further exploration on the dynamical prior and OSN responses were left for the future work.

After initialization, we run the RNN through the forward integration method discussed in Appendix F.1, and record data at some chosen sample rate into a tensor, where the further analysis and metrics are computed on.

F.4.2 Dynamics demonstration

In Figures S1 and S2, we show the dynamics of the model during the process of estimating a single group of odorants, under sparse binary and dense gamma sensing matrices respectively.

We set the the number of presented odors $n_{\text{present}} = 5$, the dictionary size $n_{\text{odor}} = 500$, and the number of sensor $n_{\text{OSN}} = 300$. Other parameters remain the same as in Table 2 except we use $\gamma = 1$ in dynamics simulation.

We set the appearance of odorants to be 0.25 sec after the network starts running. Then the odorants are appears with concentration 40 for a duration of 0.5 seconds, while the network runs for another 0.25 sec after odorants disappear. This design allows us to illustrate the baseline steady state of the network right after initialization and after odorants disappearing. We record the activities of neurons in the network with a sample rate of 1000 Hz.

In Figure 3, we show the dynamics of the model during the process of estimation two groups of odorants, under sparse binary sensing matrices. The simulation procedure remains the same, except that we generate a sensory scene that involves

bioRxiv preprint doi: https://doi.org/10.1101/2025.11.01.686013; this version posted November 3, 2025. The copyright holder for this preprint (which was not certified by peer review) is the author/funder, who has granted bioRxiv a license to display the preprint in perpetuity. It is made available under aCC-BY 4.0 International license.

independent groups of odorants with slow-changing concentration. We plot the output concentration estimate $\mathbf{c} \odot \mathbf{p}$ to illustrate the final output of our model.

We set the number of presented odors $n_{\text{present}} = 6$, arranged in two groups of three odorants. We maintain all other setting the same as in the single group dynamics simulation above.

F.4.3 Simultaneous and rapid detection capacity:

In Figures 6 and S5, we assess the model's ability to infer the presence and concentration of multiple odorants simultaneously presented.

We vary the number of presented odors n_{present} from 1 to 100, while fixing the total number of sensors $n_{\text{OSN}} = 300$ and the dictionary size. For simulation using the sparse binary sensing matrix, we let $n_{\text{odor}} = 1000$ (Figures 6 and S1). For simulation using the dense Gamma sensing matrix, we set $n_{\text{odor}} = 500$ (Figure S5). This is because using a sparse binary matrix improves the capacity of the model.

For each $1 \leq n_{\text{present}} \leq 100$, we run 40 independent simulations. In each simulation, we present n_{present} odorants to the model for an interval of length $T = 0.75s$ and record the models' estimates with a sample rate of 100 Hz. Under this setting, a slice of the recorded tensor at some timepoint t represent the estimates produced by the model when given an available time window of ts. To assess the quality of the estimates, we compute three metrics score from these slices: mean absolute error (MAE), correct proportion with tolerance δ=10, and AUROC score (defined in Appendix F.3.2). We then take the averaged scores throughout the 40 trials as the final results.

Results of this simulation are shown in Figures 6 and S5. We first focus on evaluating the concentration estimates. The averaged MAE for each $1 \leq n_{\text{present}} \leq 100$ over the time course of $T = 0.75s$ is shown as heatmaps in panel **b**. As a complement, smoothed contours of equal correct proportion are overlaid on top of the heatmaps. While MAE shows the quantitative measure of the accuracy, the correct proportion scores give more qualitative indication of the model performance. For visualization purposes, we smoothed the contours with a Gaussian filter of standard deviation $\sigma = 1$. Across heatmaps, we ensured the color axis are the same so the fair visual comparison across different conditions can be made.

Furthermore, we plot vertical slices of the heatmaps at $t_1 = 100$ms and $t_2 = 600$ms separately in panel **a**. They shows as MAE as a function of n_{present} at that two timepoint. The shaded area around the curves represents $\pm 1.96 \times SEM$, indicating 95% confidence interval of the mean.

When evaluating the presence estimates, similar procedure is repeated. The averaged AUROC score for each $1 \leq n_{\text{present}} \leq 100$ over the time course of $T = 0.75s$ is shown as heatmaps in panel **d**. Contours of equal AUROC score are overlaid. Vertical slices of the AUROC score heatmaps at $t_1 = 100$ms and $t_2 = 600$ are also plotted as curves with shaded region indicating 95%C.I. in panel **c**.

F.4.4 Scaling capacity:

In Figures 7 and S6, we aim to investigate how the number of receptor types n_{OSN} required to effectively detect a fix number of presented odors n_{present} scales with the dictionary size n_{odor}. Thus, we vary n_{OSN} linearly from 100 to 800 with an increment of 50 and sampled n_{odor} over 16 evenly spaced points in log scale ranging from 2000 to 16000. This created a 2D grid of $(\tilde{n}_{\text{OSN}}, \tilde{n}_{\text{odor}})$ pairs.

For each pair of $(\tilde{n}_{\text{OSN}}, \tilde{n}_{\text{odor}})$, we perform three independent runs of binary search to find the *maximum simultaneous detection capacity* κ defined in Appendix F.3.4, where we set the search space $S = \{n | n = 5i,\ i = 1, \dots, 20\}$. Particularly, in Figure 7, we use maximum detection capacity assessed by presence estimates κ_{AUROC} with a threshold $\epsilon_t = 0.85$; in Figure S6, we use its concentration counterpart κ_{MAE} with a threshold of 10, while the true concentration is 40. In each binary search, we ran simulations under the condition $n_{\text{OSN}} = \tilde{n}_{\text{OSN}}, n_{\text{odor}} = \tilde{n}_{\text{odor}}$, and $n_{\text{present}} = j$, where $j \in S$ is taken from the sequence (search path) $\{j_i\}_{i=1}^{k}$ produced by the binary search algorithm. The available time for estimation T is 0.2 sec in Figure 7 and 0.5 sec in Figure S6. The concentration is harder to estimate so we increase the time available.

We then used the averaged results of the three independent run, $\overline{\kappa}$, as the final estimate of the maximum simultaneous detection capacity. We repeated this process across the 15×16 pairs of $(\tilde{n}_{\text{OSN}}, \tilde{n}_{\text{odor}})$, and visualized the resulting grid of $[\overline{\kappa}_{(\tilde{n}_{\text{OSN}}, \tilde{n}_{\text{odor}})}]$ as a heatmap. Contours of equal AUROC score or MAE is further overlaid onto the heatmaps. All heatmaps within the same figures share the same color axis.

bioRxiv preprint doi: https://doi.org/10.1101/2025.11.01.686013; this version posted November 3, 2025. The copyright holder for this preprint (which was not certified by peer review) is the author/funder, who has granted bioRxiv a license to display the preprint in perpetuity. It is made available under aCC-BY 4.0 International license.

The use of binary search design significantly reduces the computational cost, without whom the experiment would be computationally infeasible, as binary search guarantees we can find the κ using at most $\lceil \log_2(|S|) \rceil = 5$ simulations, whereas a naive linear search would easily require more than 10 simulations.

Using this efficient pipeline, we are not only able to directly compare the scaling capacity of our SDEO model with the non-separated baseline model, but also investigate the effect of the affinity matrix and presence prior on the scaling capacity. We tested 9 combinations between three types of affinity matrix (Appendix E) and three types of model variants (non-separated, SDEO with Bernoulli prior and SDEO with Kumaraswamy prior).

G Fang et al.'s approach to sampling with L_0 priors in rate networks

As noted in the Discussion, Rao-Blackwellization is not the only way one can design a rate network to sample with an L_0 prior. In particular, Fang *et al.* [82] proposed a Langevin sampling algorithm for sparse coding with an L_0 prior. Here, we detail this model as applied to the olfactory sensing problem, following the discussion in Appendix E of our previous work [39]. Our starting point is a spike-and-slab prior on odor concentrations:

$$p(c_i) = \varpi e^{-\lambda c_i} + (1 - \varpi)\delta(c_i), \tag{G.1}$$

where for simplicity we will assume that *a priori* each odor is present with the same probability ϖ, and given that it is present its concentration is drawn from an exponential distribution of rate λ. Now, define an auxiliary variable $\mathbf{u}$ that is mapped to concentration estimates $\mathbf{c}$ via element-wise soft thresholding:

$$\mathbf{c} = f(\mathbf{u}), \tag{G.2}$$

where

$$f(u) = \begin{cases} 0 & u < u_0 \\ u - u_0 & u \geq u_0 \end{cases} \tag{G.3}$$

is the soft-thresholding function for threshold

$$u_0 = -\frac{1}{\lambda} \log \varpi. \tag{G.4}$$

Given an observation $\mathbf{s}$, we then run the following unconstrained Langevin dynamics for $\mathbf{u}$:

$$d\mathbf{u}(t) = \left[\nabla_{\mathbf{c}} \log p(\mathbf{s} \mid \mathbf{c}) \Big|_{\mathbf{c}=f(|\mathbf{u}|)} \odot \Theta(|\mathbf{u}| - \mathbf{u}_0) - \lambda \operatorname{sign}(\mathbf{u}) \right] dt + d\mathbf{B}(t), \tag{G.5}$$

where $\mathbf{u}_0 = u_0 \mathbf{1}$. For a Poisson likelihood as used elsewhere, we have

$$d\mathbf{u}(t) = \left[\{\mathbf{A}^\top [\mathbf{s} \oslash (\mathbf{r}_0 + \mathbf{A} f(|\mathbf{u}|)) - \mathbf{1}]\} \odot \Theta(|\mathbf{u}| - \mathbf{u}_0) - \lambda \operatorname{sign}(\mathbf{u}) \right] dt + d\mathbf{B}(t). \tag{G.6}$$

Like our model, this is a gated RNN, though of a different form.

bioRxiv preprint doi: https://doi.org/10.1101/2025.11.01.686013; this version posted November 3, 2025. The copyright holder for this preprint (which was not certified by peer review) is the author/funder, who has granted bioRxiv a license to display the preprint in perpetuity. It is made available under aCC-BY 4.0 International license.

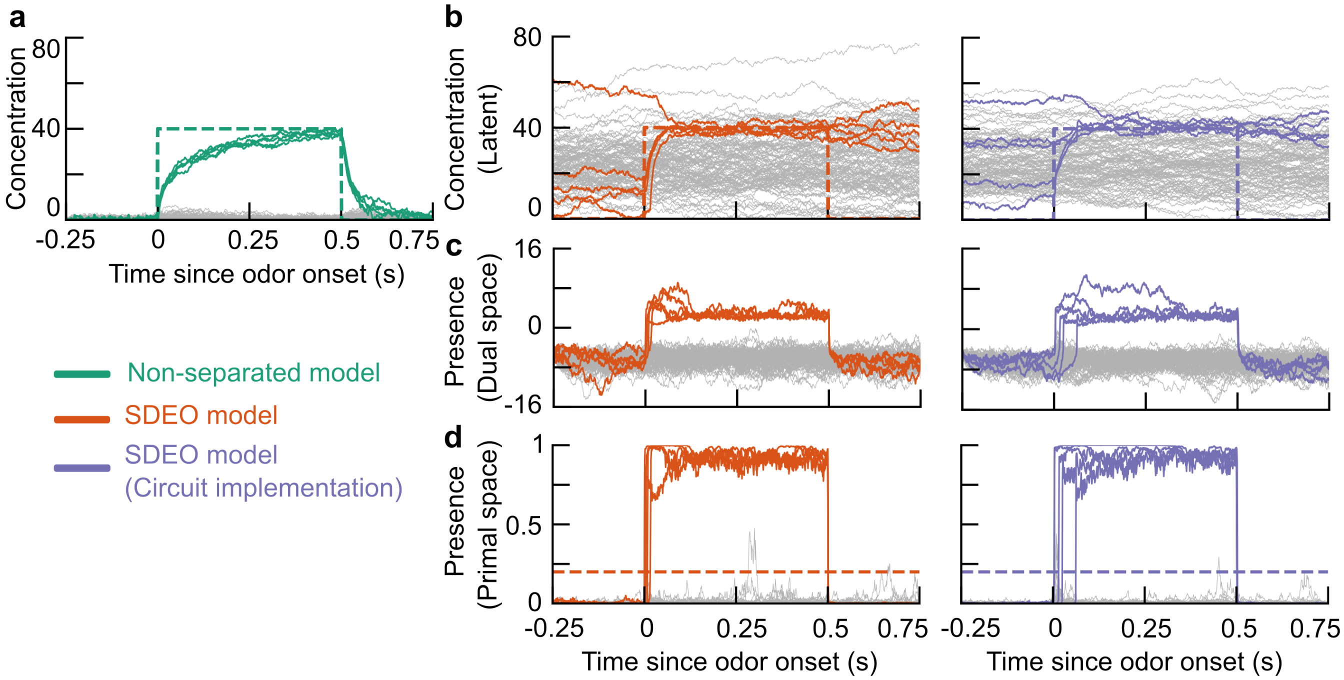

Figure S1: Dynamics of non-separated and SDEO models during the estimation process under sparse binary sensing matrices. We test three models—non-separated (as in [39]), SDEO, and SDEO with circuit implementation—on a simple estimation task to give qualitative illustration of the model dynamics during olfactory sensing. In the task, a randomly selected set of 5 out of 500 odorants appears at concentration 40 for a duration of 0.5 s. The three columns illustrate the dynamics of three models respectively, and the three rows show different quantities estimated. In each plot, the colored lines denote the values for the presented odorants, while the gray lines represent those for the background (non-presented) odorants. **a.** Estimated concentration. The dashed line traces true concentration over time. **b.** Estimated presence in the dual space. **c.** Estimated presence in the [0, 1]-bounded primal space. The dashed line marks the threshold used to binarize the presence variable during inference. Here we used sparse binary sensing matrices with sparsity 0.1 (defined in Appendix E). We ran the same simulation using dense Gamma sensing matrices and showed the results in Figure S2. For details of implementations, see Appendix F.4.2.

bioRxiv preprint doi: https://doi.org/10.1101/2025.11.01.686013; this version posted November 3, 2025. The copyright holder for this preprint (which was not certified by peer review) is the author/funder, who has granted bioRxiv a license to display the preprint in perpetuity. It is made available under aCC-BY 4.0 International license.

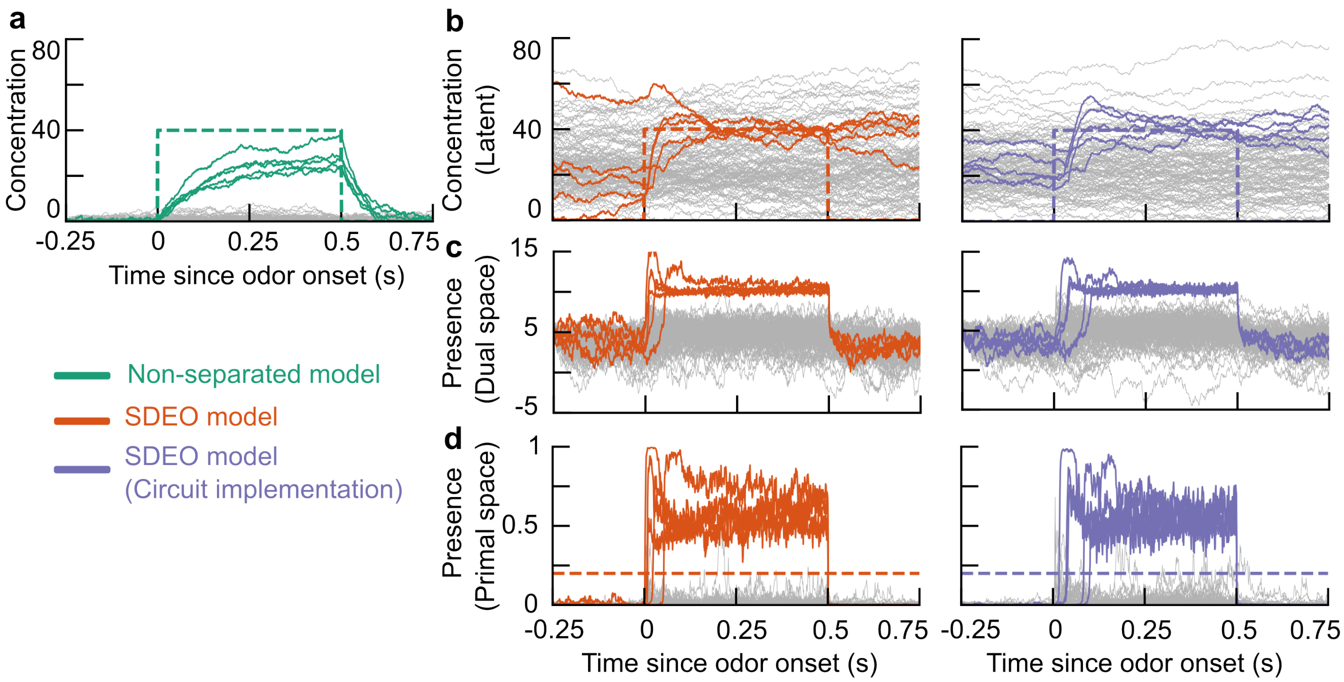

Figure S2: Dynamics of non-separated and SDEO models during the estimation process under dense Gamma sensing matrices. Here we re-ran the same simulation as in Figure S1 but used dense gamma affinity matrices instead. Hence, for details of the experiment, see the caption under Figure S1. **a.** Estimated concentration. The dashed line traces true concentration over time. **b.** Estimated presence in the mirror space. **c.** Estimated presence in the [0, 1]-bounded space. The dashed line marks the threshold used to binarize the presence variable during inference. For details of implementations, see Appendix F.4.2.

bioRxiv preprint doi: https://doi.org/10.1101/2025.11.01.686013; this version posted November 3, 2025. The copyright holder for this preprint (which was not certified by peer review) is the author/funder, who has granted bioRxiv a license to display the preprint in perpetuity. It is made available under aCC-BY 4.0 International license.

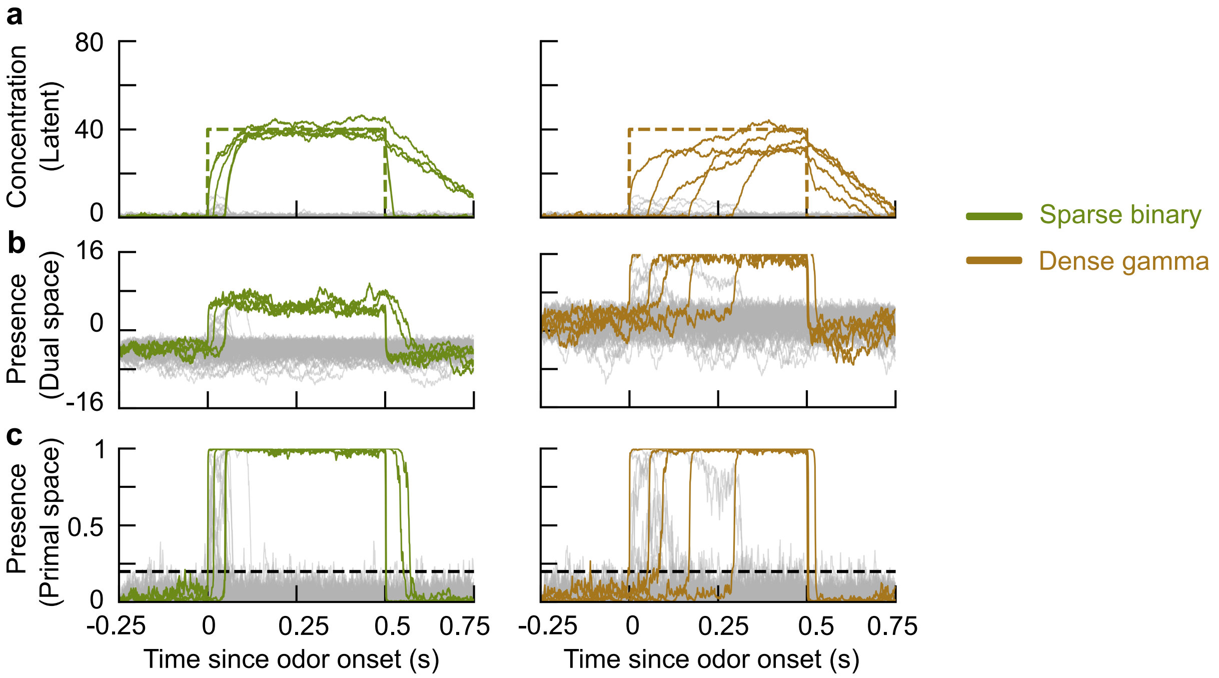

Figure S3: Dynamics of SDEO models using presence-dependent concentration priors with sparse binary and dense gamma affinity matrices. We set the rate of the exponential prior λ as 2.5. Once the odors disappear at 0.5s, the presence estimate falls below the threshold, and the exponential prior gradually suppresses the concentration estimate toward 0.

bioRxiv preprint doi: https://doi.org/10.1101/2025.11.01.686013; this version posted November 3, 2025. The copyright holder for this preprint (which was not certified by peer review) is the author/funder, who has granted bioRxiv a license to display the preprint in perpetuity. It is made available under aCC-BY 4.0 International license.

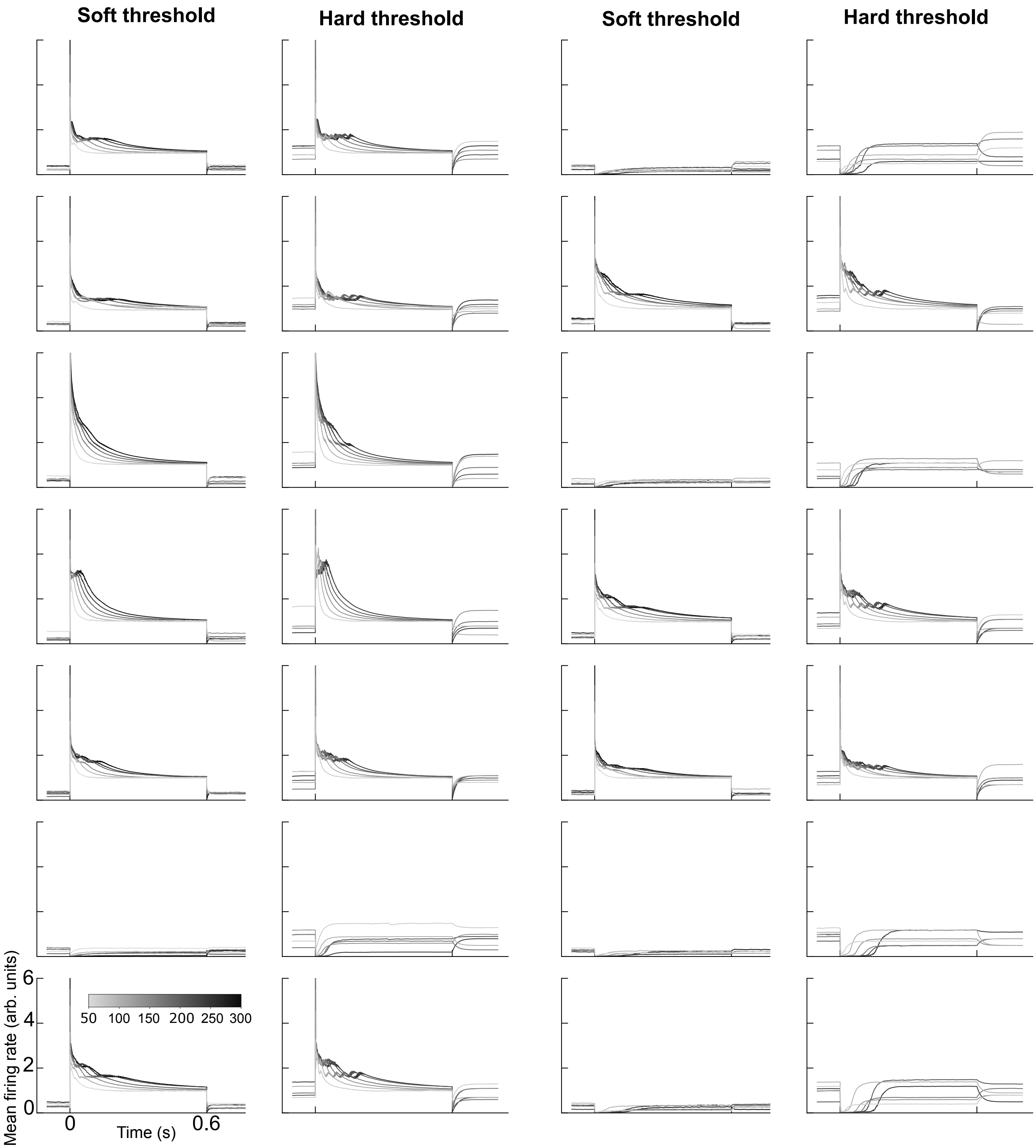

Figure S4: Responses of single projection neurons under different thresholding schemes. Each pair of panels depicts the corresponding neurons simulated with soft and hard gating, allowing direct comparison of their effects on firing dynamics. A set of fixed odor stimuli is present at 0s and withdrawn at 0.6s. Darker color indicates stronger stimulation.

bioRxiv preprint doi: https://doi.org/10.1101/2025.11.01.686013; this version posted November 3, 2025. The copyright holder for this preprint (which was not certified by peer review) is the author/funder, who has granted bioRxiv a license to display the preprint in perpetuity. It is made available under aCC-BY 4.0 International license.

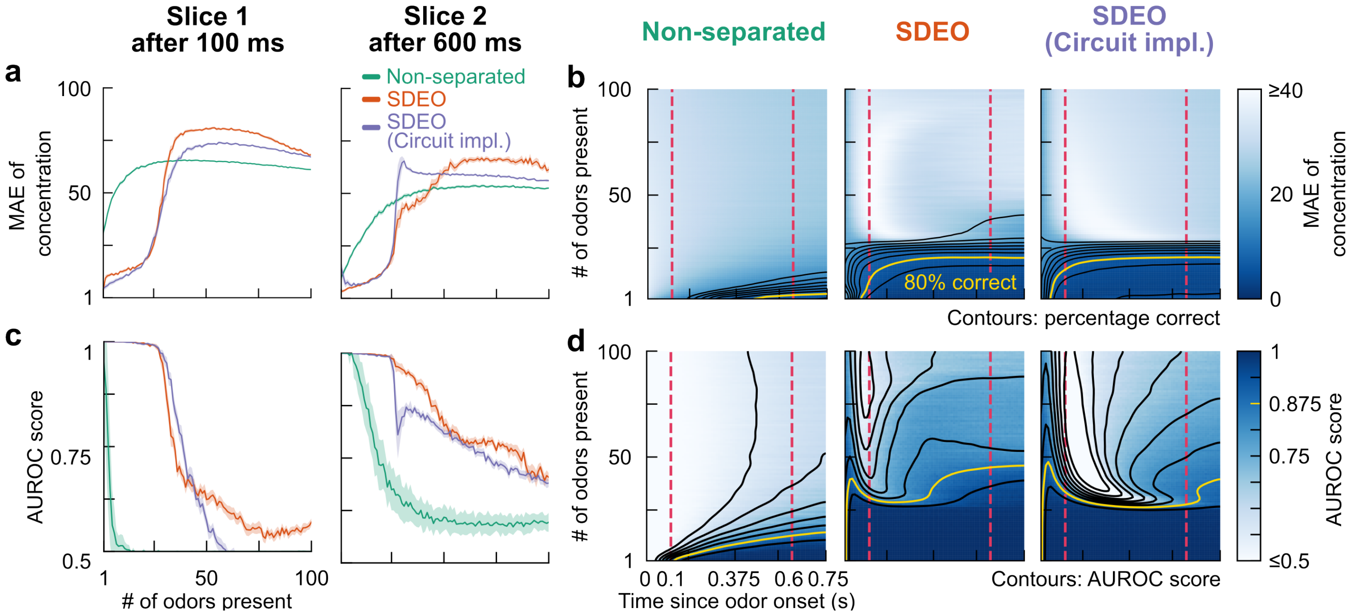

Figure S5: Improvement in fast detection of multiple odorants through separation of inference. We repeat the same simulation as in Figure 6 but using dense Gamma affinity matrices instead of sparse binary affinity matrices. We evaluate the same three models as in Figure 3 in a series of simulation where increasing numbers of odorants are simultaneously presented. In each run, in a set of 1000 odorants, a number of them are randomly selected and presented to the model for a duration of 0.75 s. We increase number of presented odors from 1 to 100, while repeat each setting for 40 times, compute the metrics and then take the average as final results. The shaded areas in **a** and **c** show $\pm 1.96 \cdot \mathrm{SEM}$ (representing 95% C.I) over realizations throughout. Row 1 assesses the models' performance in odorants concentration estimation using mean absolute error. **a.** Mean absolute error of estimated concentration as a function of the number of odorants present at two timepoints after odor onset. **b.** Heatmap of mean absolute error over inference time and number of presented odorants, with smoothed contours of correct detection fraction overlaid. Row 2 assesses the models' performance in odorants presence estimation. For the non-separated model, we convert the concentration estimation into presence estimation by binarizing the estimated concentrations based on whether they exceeds half of the true odorant concentration. Since presence estimation is a binary classification task, we use AUROC score as the performance metric. **c.** AUROC score as a function of the number of odors present at two timepoints after odor onset. **d.** Heatmap of AUROC score over inference time and number of presented odors, with smoothed contours overlaid. For details of implementation, see Appendix F.4.3.

bioRxiv preprint doi: https://doi.org/10.1101/2025.11.01.686013; this version posted November 3, 2025. The copyright holder for this preprint (which was not certified by peer review) is the author/funder, who has granted bioRxiv a license to display the preprint in perpetuity. It is made available under aCC-BY 4.0 International license.

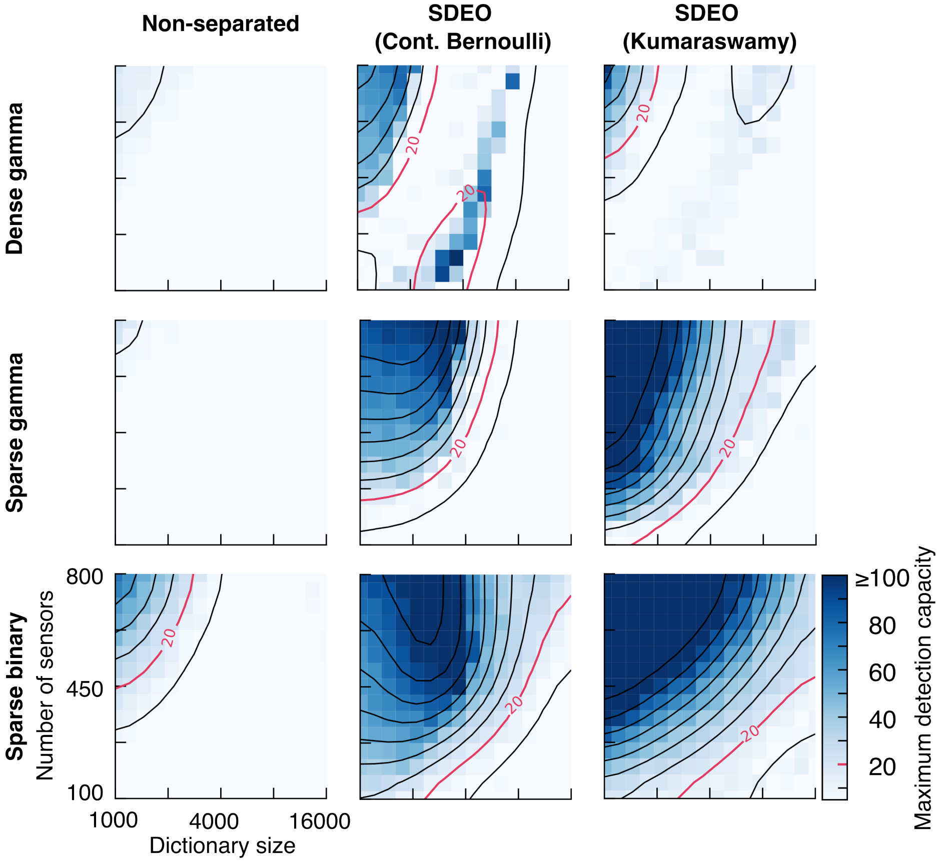

Figure S6: Scaling of detection capacity with dictionary size and sensor repertoire for different priors and sensing matrix models. We run the same simulation as in Figure 7, but showing maximum detection capacity assessed by concentration instead of presence estimates. The three columns correspond to three models—non-separated, SDEO, and SDEO with circuit implementation—and the three rows correspond to three types of affinity matrices. These are: dense Gamma, whose entries are i.i.d. random variable following $\mathrm{Gamma}(0.37, 0.36)$; sparse Gamma, obtained by applying a 0.1 sparsity mask to a dense Gamma matrix; and sparse binary, whose entries are i.i.d. random variable following $\mathrm{Bernoulli}(0.1)$. Each heatmap shows the maximum detection capacity assessed by concentration estimates for combinations of sensors counts (from 100 to 800, equally spaced linearly) and dictionary size (1000 to 16000 equally spaced on a log scale). The maximum detection capacity κ_{MAE} is defined as the largest number of simultaneously presented number of odorants that the model can detect with a mean absolute error ≤ 10, while $c_{True} = 40$. Smoothed contours are overlaid and can be interpreted as the required number of sensors to maintain a certain capacity as a function of dictionary size. The total inference time duration is 0.5 s for all runs, and the value in each cell of the heatmap is the average of three independent runs. For details of implementation, see Appendix F.4.4.

bioRxiv preprint doi: https://doi.org/10.1101/2025.11.01.686013; this version posted November 3, 2025. The copyright holder for this preprint (which was not certified by peer review) is the author/funder, who has granted bioRxiv a license to display the preprint in perpetuity. It is made available under aCC-BY 4.0 International license.

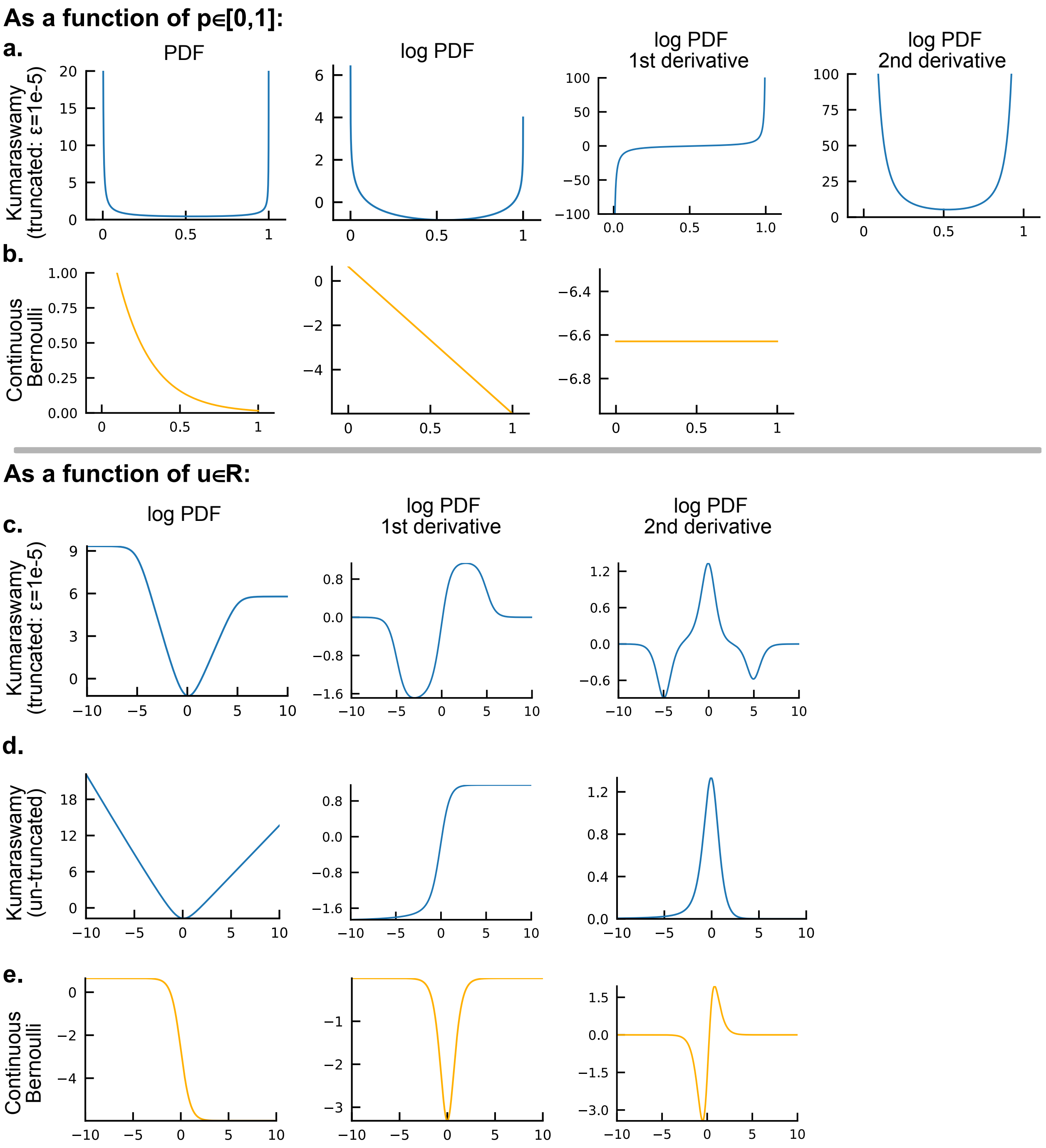

Figure S7: In **a.** and **b.**, we plot the Truncated Kumaraswamy distribution and continuous Bernoulli distribution on $[0, 1]$. **a.** Truncated Kumaraswamy distribution with parameters $a = 0.055, b = 0.422, \varepsilon = 1e - 5$. The four columns from left to right respectively are: probability density function (PDF), log PDF, the first derivative of log PDF, and the second derivative of log PDF. **b.** Continuous Bernoulli distribution with $\varpi = 0.01$. The three columns from left to right respectively are: probability density function (PDF), log PDF, and the first derivative of log PDF. We didn't show the second derivative of log PDF because it vanishes. In **c.**, **d.** and **e.** we plot three distributions with respect to $u \in \mathbb{R}$ such that $p = \frac{1}{1+e^{-\gamma u}} \in [0, 1]$. This is to illustrate the score function in the dual space. The three columns from left to right respectively are the log PDF, the first derivative of the log PDF, and the second derivative of the PDF. **c.** Truncated Kumaraswamy distribution with the same parameters as in **a**. **d.** Un-truncated Kumaraswamy distribution with parameters $a = 0.055, b = 0.422, \varepsilon = 0$. **e.** Continuous Bernoulli distribution with $\varpi = 0.01$.

bioRxiv preprint doi: https://doi.org/10.1101/2025.11.01.686013; this version posted November 3, 2025. The copyright holder for this preprint (which was not certified by peer review) is the author/funder, who has granted bioRxiv a license to display the preprint in perpetuity. It is made available under aCC-BY 4.0 International license.

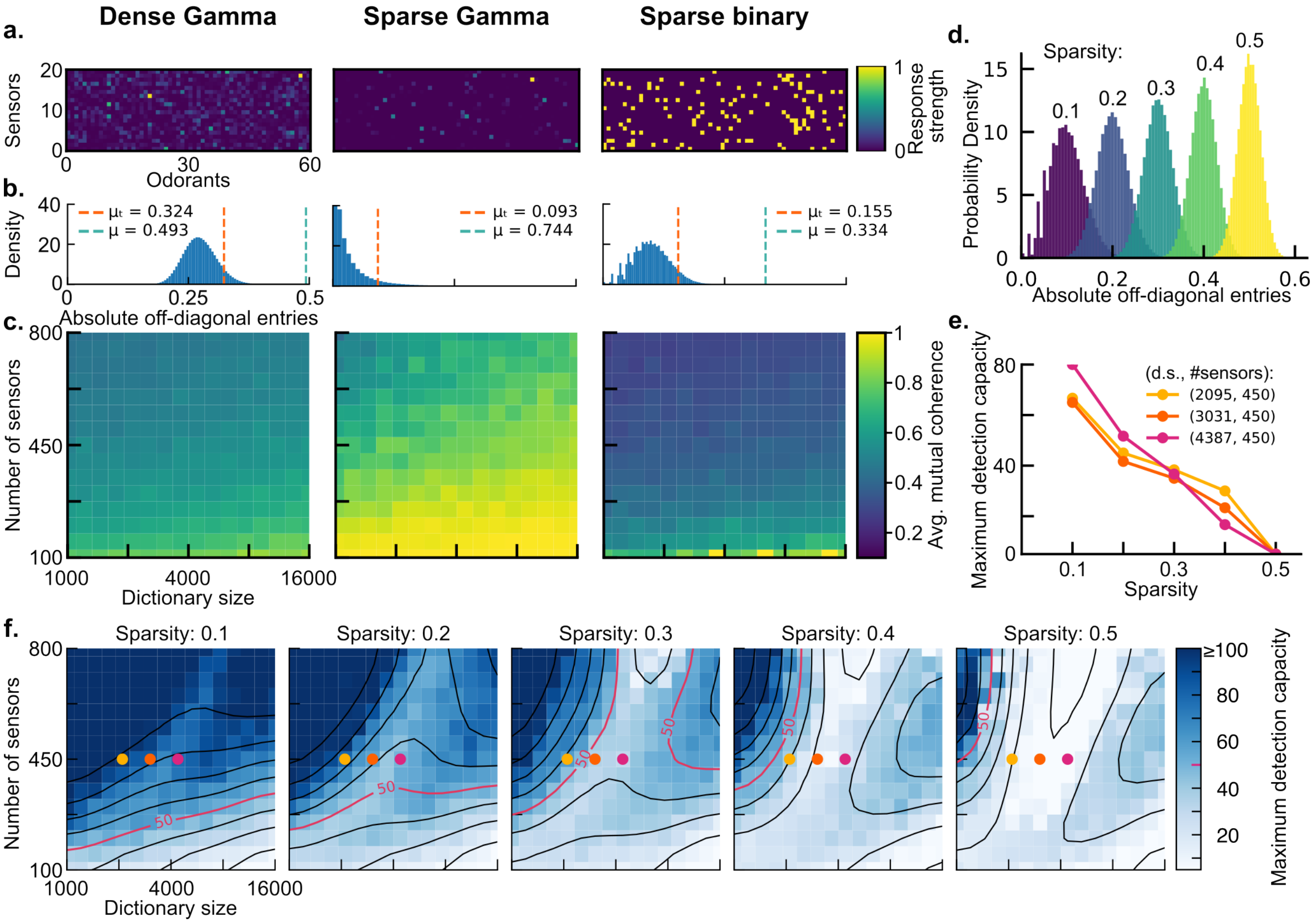

Figure S8: **a.** Zoomed in example of the three types of sensing matrix—dense Gamma with Gamma$(0.36, 0.37)$, sparse Gamma with Gamma$(0.36, 0.37)$ and sparsity 0.1, and sparse Binary with sparsity 0.1. **b.** Histograms showing the distribution of absolute off-diagonal entries of the Gram matrix of the sensing matrices, which is equivalent to column-wise correlation in the sensing matrix. Three shows distinct sensing matrix types matching the column titles. Here we use a system with 600 sensors and 5000 dictionary size. The worst case correlation μ, namely the mutual coherence, is indicated by the green vertical dashed line overlaid to the histogram, and also printed out in the legend. The average of the top 20% largest correlation μ_t, is indicated by the orange vertical dashed line overlaid to the histogram, and also printed out in the legend. **c.** Mutual coherence of the sensing matrices under different dimensionality, averaged across 5 random trials. **d.** Histogram showing the probability density of absolute off-diagonal entries of sparse binary sensing matrices with different sparsity. **e.** Maximum detection capacity as a function of sparsity of the sparse binary sensing matrix, under different combination of dictionary size and sensor repertoire. The dimensionality is indicated by dots with corresponding color in panel **f. f.** Heatmap of maximum detection capacity under different dictionary size and sensor repertoire when using sparse binary sensing matrices with increasing sparsity (s=0.1 to 0.5)
